# Ionizing radiation induces stem cell-like properties in a caspase-dependent manner in *Drosophila*

**DOI:** 10.1371/journal.pgen.1007659

**Published:** 2018-11-21

**Authors:** Shilpi Verghese, Tin Tin Su

**Affiliations:** 1 Department of Molecular, Cellular and Developmental Biology, University of Colorado, Boulder, CO, United States of America; 2 University of Colorado Comprehensive Cancer Center, Anschutz Medical Campus, Aurora, CO, United States of America; Geisel School of Medicine at Dartmouth, UNITED STATES

## Abstract

Cancer treatments including ionizing radiation (IR) can induce cancer stem cell-like properties in non-stem cancer cells, an outcome that can interfere with therapeutic success. Yet, we understand little about what consequences of IR induces stem cell like properties and why some cancer cells show this response but not others. In previous studies, we identified a pool of epithelial cells in *Drosophila* larval wing discs that display IR-induced stem cell-like properties. These cells are resistant to killing by IR and, after radiation damage, change fate and translocate to regenerate parts of the disc that suffered more cell death. Here, we report the identification of two new pools of cells with IR-induced regenerative capability. We addressed how IR exposure results in the induction of stem cell-like behavior, and found a requirement for IR-induced caspase activity and for Zfh2, a transcription factor and an effector in the JAK/STAT pathway. Unexpectedly, the requirement for caspase activity was cell-autonomous within cell populations that display regenerative behavior. We propose a model in which the requirement for caspase activity and Zfh2 can be explained by apoptotic and non-apoptotic functions of caspases in the induction of stem cell-like behavior.

## Author summary

Ionizing Radiation (IR), alone or in combination with other therapies, is used to treat an estimated half of all cancer patients. Yet, we understand little about why some tumors cells respond to treatment while others grow back (regenerate). We identified specific pools of cells within a *Drosophila* organ that are capable of regeneration after damage by IR. We also identified what it is about IR damage that allows these cells to regenerate. These results help us understand how tissues regenerate after IR damage and will aid in designing better therapies that involve radiation.

## Introduction

Regeneration is essential to tissue homeostasis and health. Conversely, regeneration of tumors after treatment leads to tumor recurrence and treatment failure. Understanding mechanisms that underlie regeneration is therefore important not only for understanding basic biology but also for optimizing treatment of diseases like cancer. Our understanding of regeneration has benefited immensely from experimental systems with dedicated stem cells that form the cellular basis for regeneration. Examples include regeneration of vertebrate gut and *Drosophila* intestine [1–3]. Tissues also regenerate despite the lack of a dedicated stem cell pool. A prime example is the vertebrate liver, which regenerates by proliferation of the surviving cells of each cell type [4–6]. If proliferation of hepatocytes is blocked during liver regeneration, however, biliary epithelial cells can dedifferentiate, proliferate and re-differentiate into hepatocytes [4–6]. Such plasticity has been documented in other mammalian organs [7–9], and in some models of amphibian limb and fish fin regeneration [10]. This report addresses the molecular basis for cell fate plasticity during regeneration using *Drosophila* larval cells as a model.

**Fig 1:**
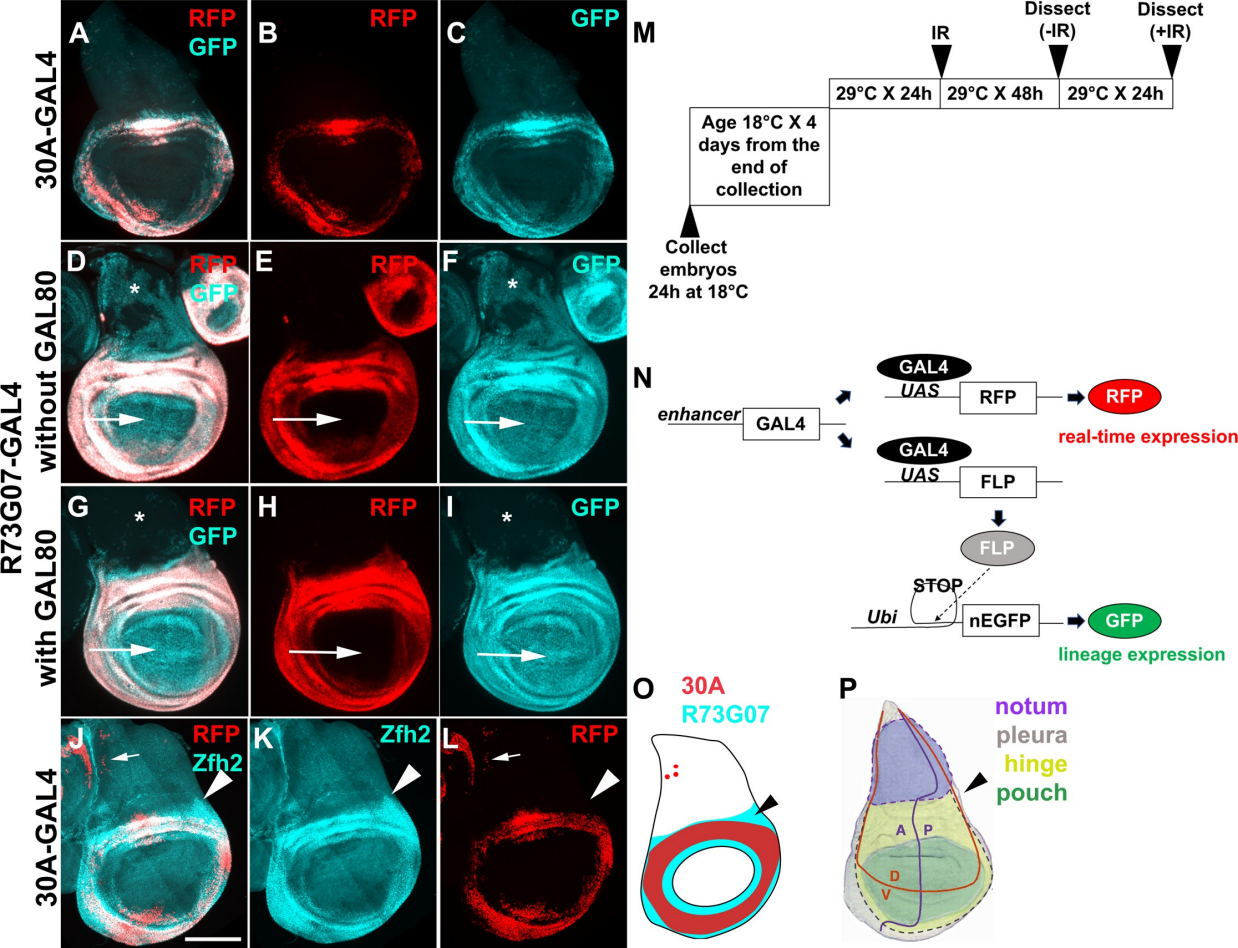
Lineage tracing with two GAL4 drivers that are active in the hinge. Wing discs were removed from 3^rd^ instar larvae without irradiation, fixed and imaged for RFP/GFP. The disc in J-L was stained with an antibody to Zfh2. All discs are shown with anterior (A) left and dorsal (D) up as in (P). Scale bar = 100 microns. (A-C) 30A-GAL4 drives UAS-RFP (real time marker) and GFP (lineage marker) (D-I) R73G07-GAL4 drives UAS-RFP (real time marker) and GFP (lineage marker). Arrows indicate the pouch area. Restricting GAL4 activity with GAL80^ts^ eliminated the expression of GFP in the notum (*) in (G-I). (J-L) Zfh2 antibody staining and 30A>RFP expression. Zfh2-expressing cells outside the 30A domain are indicated with arrowheads. (M) The temperature shift protocol. The embryos were collected at 18°C for 24 h and cultured at 18°C until 4–5 days after egg laying, reaching late 2^nd^ instar. The larvae were shifted to 29°C for 24 h to reach early 3^rd^ instar before irradiation with 0 or 4000 R of X-rays. The discs were dissected 48 h later (for–IR controls) or 72 h after IR (+IR samples) because IR delays development. (N) A schematic diagram to explain G-trace. Ubi = Ubi^p63E^ promoter. (O-P) Summary of 30A-GAL4 and R73G07-GAL4 expression (O) and the fate map with dotted lines added to indicate the pleura (P). Arrowheads point to the region that is R73G07-GAL4-expressing but outside the 30A domain. (P) is modified from [59]. The genotypes were: (A-C) 30A-GAL4, UAS-G-trace (see S1 Table for G-trace genotype)/SM5 (D-F) UAS-G-trace/+; R73G07-GAL4/+ (G-I) UAS-G-trace/+; R73G07-GAL4/tub-GAL80^ts^ (J-L) 30A-GAL4, UAS-G-trace /+; tub-GAL80^ts^/+.

*Drosophila* larval imaginal discs are precursors of adult organs. Imaginal discs lack a dedicated stem cell pool yet can regenerate fully even after surgical ablation of 25% of the disc, after genetic ablation of a disc compartment (e.g. by expressing a pro-apoptotic gene in the anterior compartment), or after exposure to doses of ionizing radiation (IR) that kills about half of the cells [11, 12]. We recently identified a previously unknown mode of regeneration in *Drosophila* larval wing discs, whereby epithelial cells acquire stem cell-like properties during regeneration after damage by IR [13]. These properties include resistance to killing by IR, the ability to change cell fate, and the ability to translocate to areas of the wing disc with greater need for cell replenishment. The ability to behave like stem cells in response to IR is limited to certain cells within the continuous epithelium of the wing disc. Specifically, a subset of future hinge cells (see Fig 1P for the fate map in the wing disc) is protected from IR-induced apoptosis by the action of STAT92E (*Drosophila* STAT3/5, to be called ‘STAT’ hereafter) and by Wg (*Drosophila* Wnt1)-mediated repression of pro-apoptotic gene *reaper* [13]. These hinge cells lose the hinge fate and translocate to the pouch region that suffers more apoptosis and participate in regenerating the pouch. Without IR, these cells differentiate into the adult wing hinge, indicating that cell fate plasticity is IR-induced.

In above-described studies, regeneration of the pouch by the hinge was observed in nearly all irradiated discs [13, 14]. In about 20% of irradiated discs, we observed, in addition, abnormal regeneration that produced an ectopic wing disc [14]. Ectopic discs were wing discs based on staining for the protein markers Ubx and Wg, and were composed of an ectopic pouch and an ectopic hinge [14]. Ectopic discs were neither duplications (e.g. not pouch-to-pouch) nor transdeterminations (e.g. not leg-to-wing) described in classical studies of regeneration after surgical ablation [15]. Our efforts to dissect the cellular origin for the ectopic discs showed that cells of the hinge that regenerate the pouch are unlikely to be responsible for ectopic discs [14]. Therefore, we hypothesized that there are additional pools of cell in the wing disc that show stem cell like properties after IR damage by participating in abnormal regeneration to produce an ectopic wing disc.

Here, we report the mapping of cell lineages during regeneration of *Drosophila* larval wing discs following damage by X-rays, a type of IR. To express lineage tracers, we used FlyLight GAL4 drivers that display simple expression patterns because they use small (~3kb) enhancers from various genes [16]. In addition to the subset of future hinge cells we previously identified as capable of behaving like stem cells [13], two more cell populations, in the notum and in the dorsal-posterior hinge/pleura region, were found to show this potential. While the previously identified hinge cells are responsible for normal regeneration to restore the wing disc, the newly identified regenerative cells undergo abnormal regeneration to produce ectopic discs. Cells of the pouch, we find, lack the capacity for plasticity and do not change fate or translocate. Of possible consequences of X-ray exposure, we identified caspase activity as an essential determinant for inducing stem cell-like properties, and further localize this requirement to the regenerative cells. Zfh2, a transcription factor and a STAT effector, we found, was needed to regulate IR-induced caspase activity and for fate change. Cancer treatments including IR induce stem cell-like properties in non-stem cancer cells [17–20], but we understand little about what consequences of IR induces stem cell-like properties. This report describes similar phenomena in *Drosophila* cells and offers molecular insights into IR-induced cell plasticity.

## Results

We are using the published G-trace system to monitor cell lineages in the larval wing discs [21]. In this system, GAL4 drives the expression of UAS-RFP (real time expression, Fig 1N). GAL4 also drives the expression of UAS-FLP recombinase, which causes a recombination event to excise transcription ‘STOP’ sequences, resulting in stable GFP expression (lineage expression). Thus, even if cells change fate and lose RFP expression, their clonal descendants would be marked with GFP (becoming GFP^+^RFP^*-*^). We used G-trace to test a collection of GAL4 drivers, each active within a different subset of cells in *Drosophila* 3^rd^ instar larval wing discs.

### Optimization of the lineage tracing protocol

30A-GAL4 was used in our recent studies and is active in a subset of hinge cells and a few (fewer than 10) of notum cells (Fig 1A–1C and 1J–1L, arrows point to RFP^+^ notum cells; [13, 14]). The dynamics of 30A-GAL4 activity is such that cells expressing it show stable lineage; very few were GFP^+^RFP^*-*^. In contrast, another hinge driver, FlyLight R73G07-GAL4 produced GFP^+^ cell in most of the disc including the pouch, the hinge, and most of the notum, even though RFP is restricted to the hinge in 3^rd^ instar wing discs (Fig 1D–1F). R73G07 is a 3028 bp enhancer from the *zfh2* locus and is apparently active in most cells of the wing disc before becoming restricted to the hinge. Zfh2 is a transcription factor important for wing development [22]. Temporal restriction of R73G07-GAL4 activity with repressor GAL80^ts^ according to the temperature shift protocol shown in Fig 1M confined GFP to the hinge and the pouch (Fig 1G–1I). Increasing larval age from 4–5 days to 5–6 days after egg deposition (AED) before temperature shift to induce GAL4 did not restrict the GFP^+^ domain further. Aging the larvae beyond 5–6 days AED before inducing GAL4 may help eliminate GFP in the pouch and restrict it to the hinge, but this schedule is incompatible with our goal because we need to monitor regeneration for 72 h after IR before losing the larvae to pupariation.

These results illustrate that while some GAL4 drivers show stable lineage expression and could be used to monitor fate changes after irradiation, others show lineage changes without IR. This was confirmed using fifteen additional FlyLight GAL4 drivers (S1 Fig). Therefore, we used GAL80^ts^ and the protocol shown in Fig 1M in all subsequent experiments to identify regenerative cell populations, even if their lineages were stable as in the 30A-GAL4 example. Although we selected FlyLight drivers with apparently exclusive expression in the disc region of interest (https://flweb.janelia.org/), many, we found, show additional expression elsewhere in the wing disc and are unsuitable for lineage tracing (S1 Fig). Some GAL4 drivers were active in the cells of the peripodial membrane that covers the wing disc epithelium on the apical side, and in wing-disc associated tracheal cells on the basal side. In such cases, peripodial and tracheal cells could be identified based on their larger nuclear size compared to columnar epithelial cells and on their location in optical sections that book-end the columnar epithelium (S2 Fig). Our analyses focused on the columnar epithelium by excluding other optical sections.

Antibody staining shows that Zfh2 protein expression resembles R73G07-GAL4>RFP expression (compare Fig 1H and 1K). In contrast, 30A>RFP is expressed in only a subset of these cells (compare Fig 1K and 1L). Of relevance to subsequent sections is the expression of R73G07-GAL4 but not 30A-GAL4 in the dorsal-posterior hinge and the pleura (arrowheads in Fig 1J–1L and 1O–1P).

### Cells of the dorsal-posterior hinge and pleura change fate and translocate into the notum

**Fig 2:**
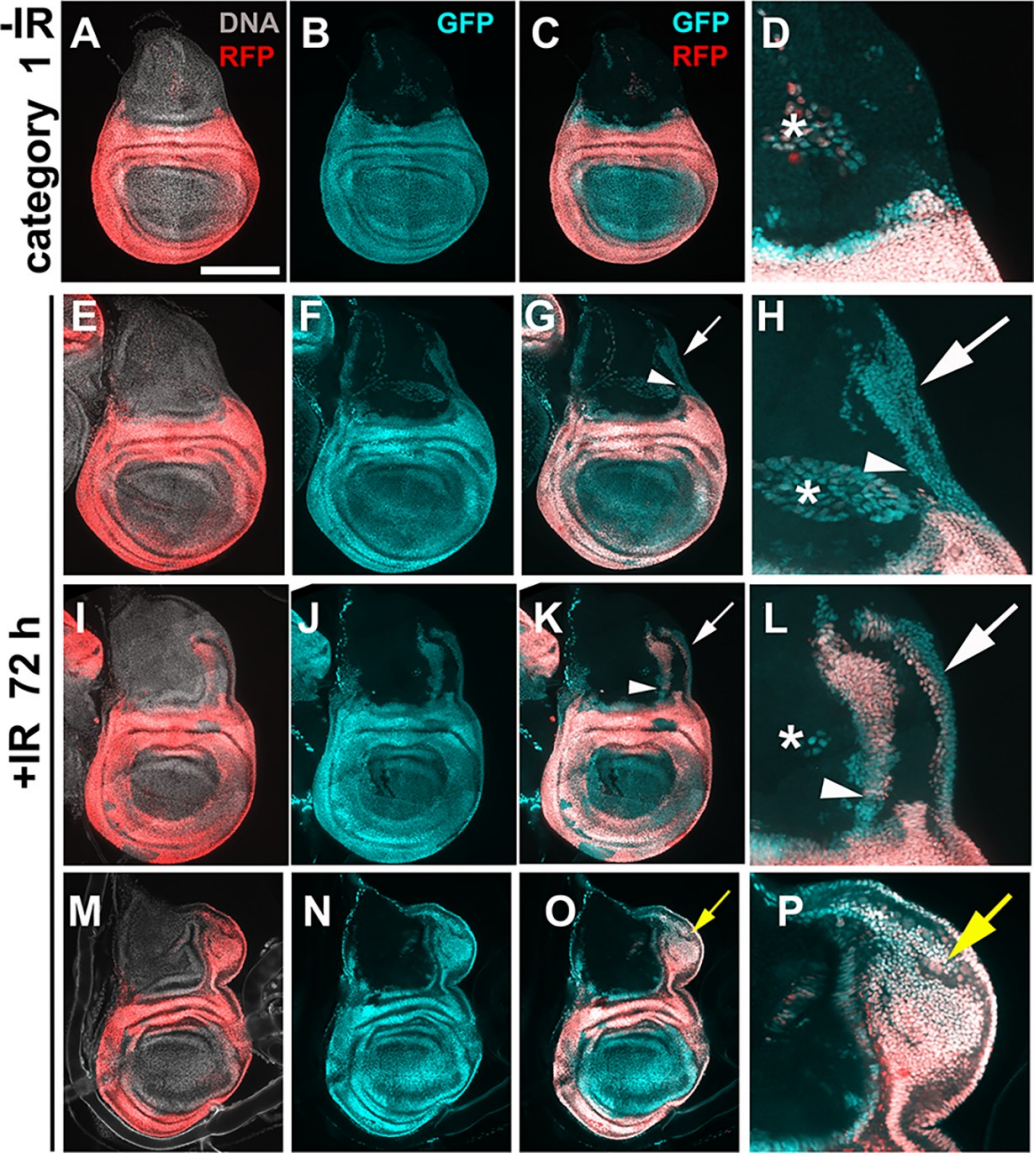
Posterior-dorsal hinge cells translocate towards the notum after IR. Larvae of the genotype UAS-G-trace/+; GAL80^ts^/ R73G07-GAL4 were treated as in Fig 1M. Wing discs are removed, fixed and imaged for RFP/GFP. All discs are shown with anterior left and dorsal up. Scale bar = 33 microns in D, H, L and P and 100 microns in the rest of the panels. Arrowheads = GFP^+^ cell populations in the notum that are contiguous with the hinge. White arrows = GFP^+^ cell populations in the notum that are contiguous with the pleura. Yellow arrows = ectopic discs. * = cells outside the columnar epithelial layer that express G-trace.

We used 4000R of X-rays for experiments and analyzed regenerated discs 72 h after irradiation. This level of IR kills more than half of the cells but the discs could still regenerate to produce viable adults [11, 12]. In our published studies of lineage tracing after irradiation, 30A-GAL4 expressing hinge cells translocated to the pouch but showed little movement dorsally towards the notum [13, 14]. In contrast, R73G07-GAL4>G-trace experiments show GFP^+^ cell populations that extend from the hinge dorsally along both the anterior and posterior margins of the wing disc (Fig 2). The extending GFP^+^ cell population is contiguous with both the hinge (arrowheads) and the pleura (white arrows, Fig 2F–2H and 2J–2L). Some GFP^+^ cells in the notum lack RFP (for example, Fig 2H arrow) while others express RFP (for example, Fig 2L arrow). In discs that show an ectopic disc (Fig 2M–2P, yellow arrows), many cells of the ectopic disc are GFP^+^RFP^+^. To better understand the source of GFP^+^ cells in the notum, we repeated the experiment but analyzed the discs at different times after IR.

**Fig 3:**
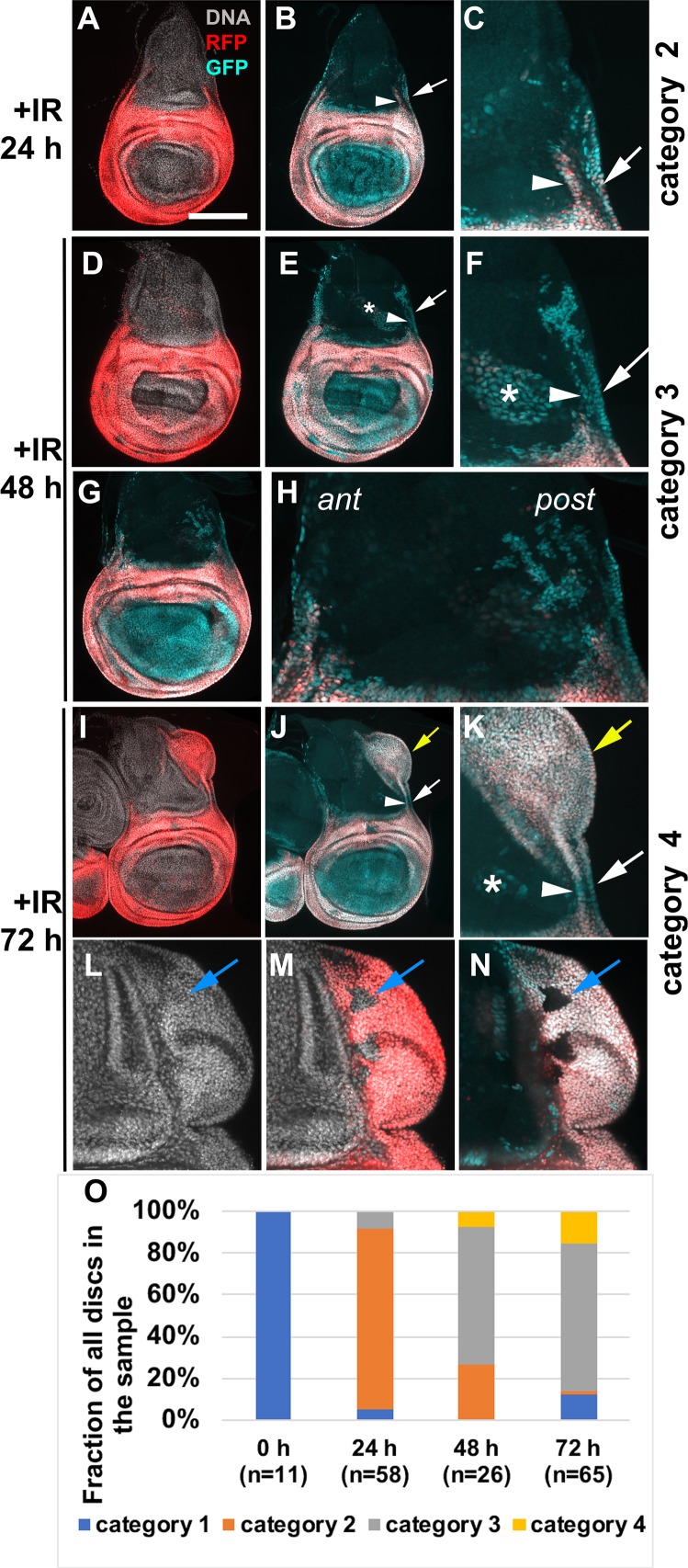
R73G07-GAL4>G-trace in a time course. Larvae of the genotype UAS-G-trace/+; GAL80^ts^/ R73G07-GAL4 were treated as in Fig 1M. Wing discs are removed, fixed and imaged for RFP/GFP at 24, 48 and 72 h after IR. The discs were also stained for DNA. All discs are shown with anterior left and dorsal up. Panels C, F, H and K show magnified portions of the disc preceding it. L-N show the ectopic disc from another 72 h disc. Arrowheads = GFP^+^ cell populations in the notum that are contiguous with the hinge. White arrows = GFP^+^ cell populations in the notum that are contiguous with the pleura. Yellow arrows = ectopic discs. Blue arrows = cells within ectopic discs that lack GFP or RFP. * = cells outside the columnar epithelial layer that express G-trace. (O) The disc were categorized according to the extent of GFP/RFP cells in the notum as described in the text; for example, Fig 2A–2D for category 1, Fig 3A–3C for category 2, Fig 3D–3H for category 3 and Fig 3I–3K for category 4. n = the number of discs analyzed for each time point, in two independent cohorts of larvae. Scale bar = 33 microns in C, F, H, K, L-N, and 100 microns in the rest of the panels.

We analyzed R73G07>G-trace larvae from the same cohort at 24, 48 and 72 h after IR, in two independent time course experiments (Fig 3). Wing discs, we found, fell into four categories depending on the abundance and location of GFP/RFP cells in the notum. Un-irradiated discs showed very few GFP/RFP cells in the notum (Fig 2A–2D, ‘category 1’). Category 2 discs showed GFP^+^RFP^+^ cells spreading dorsally from the hinge (arrowhead) and the pleura (arrow, Fig 3A and 3B, magnified in C). Category 2 predominates at 24 h after IR (Fig 3O). The presence of RFP in the hinge cells that had spread into the notum could be due to the persistence of GAL4, RFP, or both. The movement of hinge/pleura cells was seen along both the anterior and the posterior disc margins, but not in the central portion of the disc (Fig 3B). In category 3 discs, GFP^+^ cells in the notum increased in number compared to category 2, were found deeper (more dorsal) in the notum, and most lacked RFP (Fig 3E–3H). Category 3 predominated at 48 h after IR (Fig 3O). We interpret these data to mean that cells continued to translocate into the notum from the R73G07-GAL4 domain between 24 and 48 h after IR, with many terminating R73G07>RFP expression, indicative of fate change. As in category 2 discs, GFP^+^ cell population in the notum of category 3 discs appeared contiguous with both the hinge (arrowhead) and the pleura (arrow, Fig 3E, magnified in F). Moreover, GFP^+^ cells in the notum were more numerous in the posterior half (post) than in the anterior half (ant) in some discs (Fig 3G, magnified in H), which may explain the finding that ectopic discs seen at 72 h after IR always appear along the posterior wing margin (e.g. Fig 2M–2P; [14]). In category 4 discs, GFP+ cells in the notum were more numerous and more dorsal than in category 3, and most expressed RFP. In some category 4 discs, GFP^+^RFP^+^ cells were contained within the notum (similar to Fig 2I–2L) while in others GFP^+^RFP^+^ cells were in an ectopic disc (Fig 3I–3N, similar to Fig 2M–2P). Category 3 still predominated at the 72 h time point but the fraction of category 4 increased between 48 and 72 h after IR in both time courses (Fig 3O). Likewise, ectopic discs appeared between 48 and 72 h after IR, which agrees with our published results using a different GAL4 driver [14]. RFP^+^GFP^+^ cells of the ectopic discs appear contiguous with cells of the hinge and the pleura (arrowhead and arrow, respectively, in Fig 3I–3K).

How were GFP^+^RFP^+^ cells in the notum of category 4 discs produced? There are three possibilities. First, they are translocated hinge/pleura cells that never lost their original fate. Second, they are GFP^+^RFP^-^ cells in class 3 discs that re-gained their hinge/pleura fate to re-express RFP. Third, they formed de novo and bear no relation to the hinge/pleura of the primary disc. The finding that GFP^+^RFP^+^ cells in the notum appear contiguous with the primary hinge and the pleura makes us favor the first two possibilities, which are not mutually exclusive. In our published time courses, we never saw cells of the 30A domain spreading into the notum [13, 14]. We interpret these data to mean that hinge cells that translocate into the notum originate from part the hinge outside the 30A domain (arrowheads in Fig 1J–1L and 1O).

### Cells of the notum contribute to the ectopic disc

**Fig 4:**
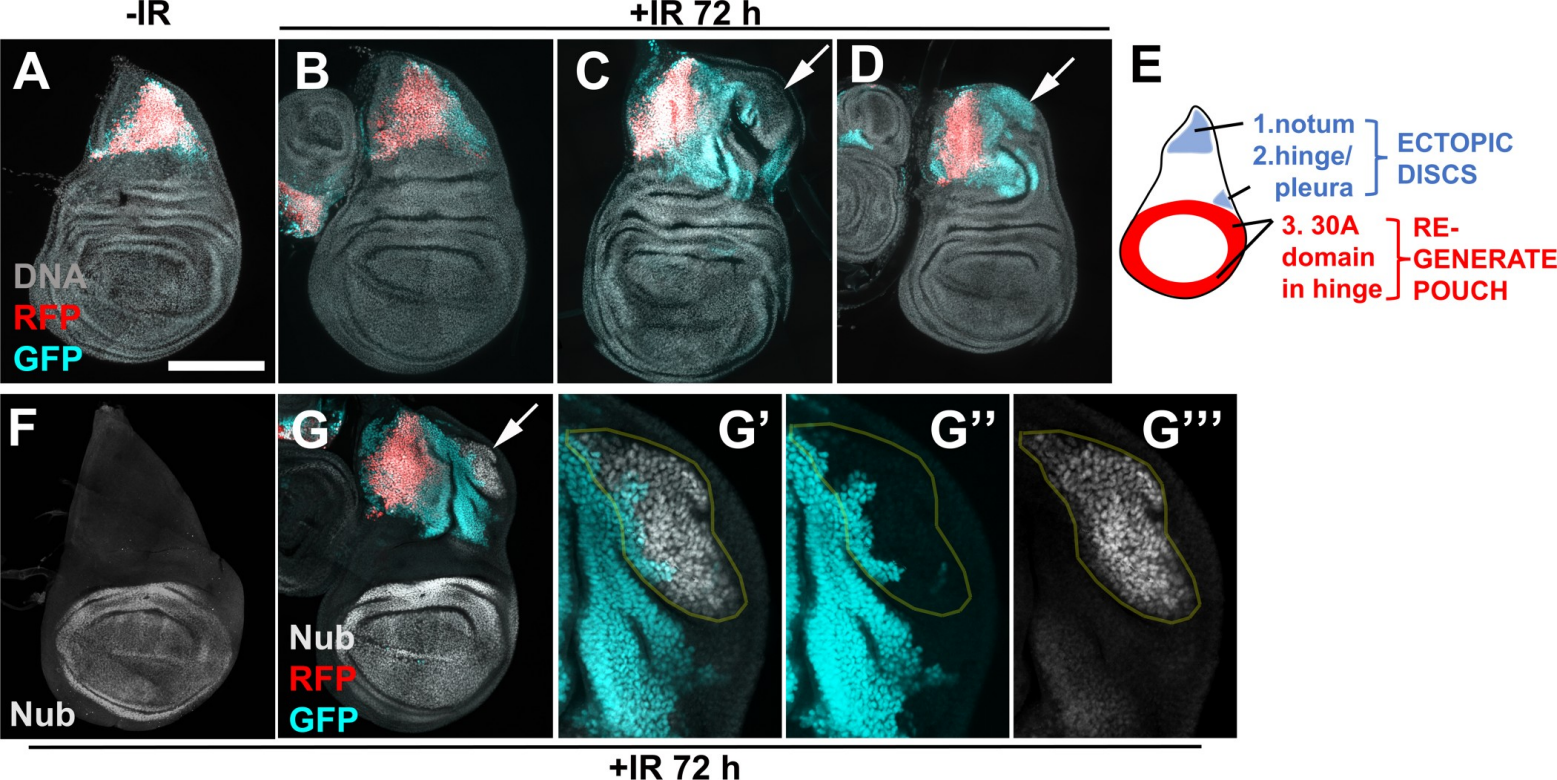
Cells of the notum contribute to the ectopic disc. Larvae of the genotype UAS-G-trace/+; GAL80^ts^/ R76A01-GAL4 were treated as in Fig 1M. Wing discs were removed, fixed and stained for DNA, and imaged for RFP/GFP. The discs in F-G were also stained with an antibody to Nubbin. All discs are shown with anterior left and dorsal up. Arrows point to ectopic discs. Some cells in the ectopic discs were GFP^-^RFP^-^ (C) and some were GFP^+^RFP^-^ (D and G). The pouch marker Nubbin was confined to the primary pouch in discs without ectopic discs (F) but appeared in some cells of the ectopic disc (G, magnified in G’-G”‘). Some Nub-expressing cells were GFP^+^ former notum cells, indicating notum to pouch fate change (circled in G’-G”‘). Scale bar = 33 microns in G’-G”‘ and 100 microns in other panels. (E) shows the location of three cell populations that change fate and/or location after irradiation.

Confocal imaging and close inspection of each optical section showed that ectopic discs included cells that lacked both RFP and GFP (Fig 3L–3N, arrows). In these experiments, the only cells that lacked RFP and GFP were notum cells, suggesting that cells of the notum also contribute to ectopic discs. We addressed this possibility directly by lineage-tracing with a notum-specific GAL4 driver (Fig 4).

R76A10-GAL4, bearing an enhancer fragment from the *tailup* locus, is active exclusively in a subset of notum cells (Fig 4A). Without IR, cell fate in this domain was stable as seen by GFP/RFP overlap (Fig 4A). At 72 h after IR, GFP/RFP overlap looked similar to–IR in most discs (Fig 4B). The exceptions were irradiated discs with ectopic growths, where we observed an expansion of the GFP^+^ cell population beyond the RFP^+^ area (Fig 4C, 4D and 4G). The lack of RFP in these cells suggests that they had lost their original fate as detected by R76A10-GAL4>RFP expression. Such GFP^+^RFP^-^ cells were part of the ectopic disc, although the extent of their contribution to the ectopic disc and their location within the ectopic disc varied from disc to disc (arrows in Fig 4C, 4D and 4G). Nubbin is a pouch marker that is not normally expressed in the notum (Fig 4F). Some GFP^+^RFP^-^ former notum cells in ectopic discs expressed Nub (Fig 4G–4G”‘), indicating that notum-to-pouch fate change occurred as these cells participated in the formation of an ectopic disc.

In our previous studies, cells of the pouch, marked with rn-GAL4>G-trace, did not change fate or translocate after irradiation [13]. Even in experiments when we directed cell death to the hinge and left the pouch cells alive, the hinge was repaired with the hinge cells and not the pouch cells [13]. We confirmed these findings in new experiments with rn-GAL4 as well as two additional pouch drivers, R42A07-GAL4 (from the *dve* locus) and R85E08-GAL4 (from the *salm* locus) (S3 Fig).

**Fig 5:**
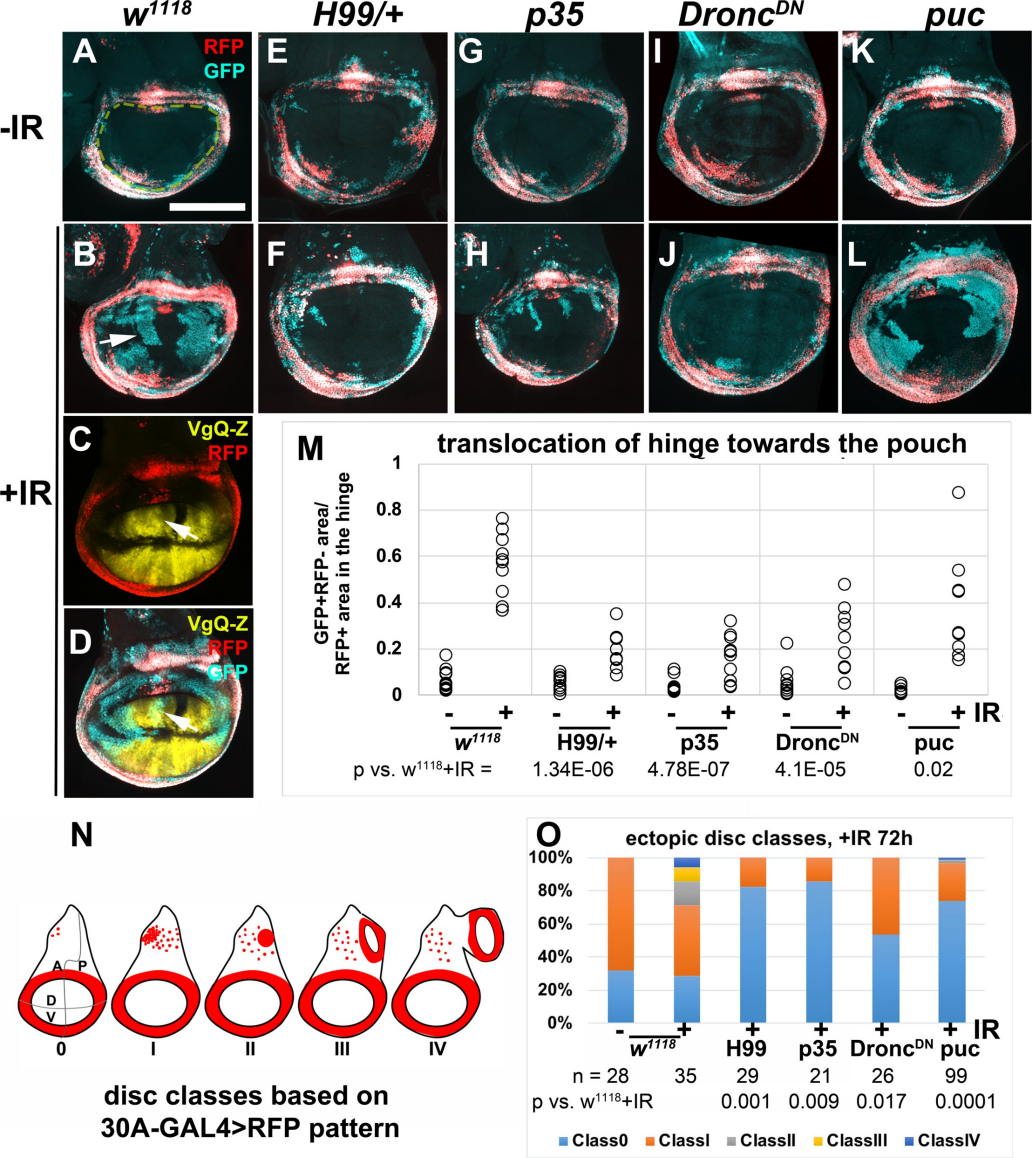
Apoptosis/caspase activity is needed for IR-induced regenerative behavior. Larvae were treated as in Fig 1M. Wing discs are removed, fixed and imaged for RFP/GFP. All discs are shown with anterior left and dorsal up. (A-B) Former hinge cells lose RFP (arrow in B) and translocate towards the pouch. (C-D) Translocated hinge cells gain the pouch fate as detected by Vestigial Quadrant Enhancer-lacZ (VgQ-Z). Arrows point to GFP^+^ former hinge cells that express VgQ-Z. (E-L) Representative wing discs from -IR (top row) and +IR (bottom row) samples. See below for genotypes. (M) GFP^+^RFP^-^ area within the pouch region (enclosed by yellow dashed line in A) was quantified in Image J and normalized to the RFP^*+*^ area of the hinge, and plotted for each genotype/condition. n = 9 for H99/+ +IR and 10 each for all other samples, from two biological replicate experiments. p-values for +IR samples were computed using 2-tailed student’s t-test. (N) Ectopic discs classes as determined by the appearance of 30A-GAL4>RFP in the notum as described before [14]; classes II-IV are IR-induced. (O) The graph shows the frequency of ectopic disc classes in experiments in (A-L). The number of discs examined for each genotype (n) is shown, along with the p-value vs. *w*^*1118*^ +IR by Fisher Exact Test (see Methods). Scale bar = 100 microns. The genotypes were: (A-B) *w*^*1118*^ = 30A-GAL4, UAS-G-trace/+; tub-GAL80^ts^/+, from a cross of *w*^*1118*^ to 30A-GAL4, UAS-G-trace/CyO-GFP; tub-GAL80^ts^/ tub-GAL80^ts^ (C-D) 30A-GAL4, UAS-G-trace/ VgQ-lacZ; tub-GAL80^ts^/TM6 (E-F) H99/+ = 30A-GAL4, UAS-G-trace/+; tub-GAL80^ts^/H99 deficiency (G-H) p35 = 30A-GAL4, UAS-G-trace/+; tub-GAL80^ts^/UAS-p35 (I-J) Dronc^DN^ = 30A-GAL4, UAS-G-trace/+; tub-GAL80^ts^/ UAS-Dronc^DN^ (K-L) puc = 30A-GAL4, UAS-G-trace/+; tub-GAL80^ts^/UAS-puc.

Collectively, the data in Figs 2–4 show that within a wing disc, cells in the hinge, the pleura and the notum can change fate and translocate after irradiation. Of these, hinge cells in the 30A-GAL4 domain translocate and change fate in nearly all irradiated discs to regenerate the pouch ([13]; see also Fig 5). In contrast, hinge cells outside the 30A domain, pleura and notum cells produce an ectopic disc in a fraction of irradiated discs (summarized in Fig 4E).

*Drosophila* wing disc is sub-divided into compartments, Anterior/Posterior and Dorsal/Ventral, for example, with cell lineages restricted to each compartment during development. During regeneration after ablation of a specific compartment, compartment boundaries collapse and are rebuilt, with some cells switching compartment identities [23]. In these models, one compartment suffered massive damage while the others were untouched. In contrast, damage by IR is scattered throughout the disc. Using the same compartment-specific GAL4 drivers as in the published study, ci-, ap-, and en-GAL4, we found that compartment boundaries remained intact in the primary disc during regeneration after IR damage (S4 Fig). In the same experiments, ectopic discs showed fluid compartment boundaries (further discussed in DISCUSSION).

### Caspase activity and/or cell death are required for IR-induced cell fate plasticity

Regenerative cells proliferate, change fate, and change location, in order to rebuild damaged tissue. Two aspects of regenerative behavior studied here, cell fate change and translocation, do not occur without IR (for example, Fig 1A and Fig 4A). Therefore, we next addressed how IR exposure is linked to these two aspects of regenerative behavior. IR has many effects on cells including DNA breaks, cell cycle arrest by checkpoints, and apoptosis. Of these, apoptosis has been demonstrated to induce proliferation of the surviving cells in a phenomenon known as Apoptosis-induced-Proliferation or AiP (reviewed in [24]). Therefore, we investigated whether apoptosis is also required for the induction of cell fate change and translocation after IR in the primary disc and for the formation of ectopic discs. In these experiments, we used 30A-GAL4>G-trace (expression pattern in Fig 1A–1C and 1J–1L, Fig 5A), which we used previously to show that former hinge cells lose GAL4 expression (become GFP^+^RFP^-^) and translocate towards the pouch ([13]; arrow in Fig 5B, which is from new experiments that reproduced the published findings). We confirmed that such cells gain the pouch fate as detected by Vestigial Quadrant Enhancer-lacZ or VgQ-Z (Fig 5C and 5D, arrows point to GFP^+^ former hinge cells that express VgQ-Z; [25]). We showed previously that regenerative behavior of the hinge cells could be quantified by measuring the GFP^+^RFP^-^ area inside the 30A circle (Fig 5A, quantified in M; see figure legend and [13] for quantification method). We showed previously that ectopic disc formation could be quantified by classifying the discs according to 30A-GAL4>RFP pattern (Fig 5N; see figure legend and [14] for quantification method). As reported previously, unirradiated *w*^*1118*^ controls show classes 0 and I only; ectopic disc classes II-IV are IR-induced ([14]; Fig 5O).

Chromosome deficiency H99 deletes several genes including three pro-apoptotic genes, *hid*, *rpr* and *skl*; H99 heterozygous wing discs show delayed and reduced IR-induced caspase activation and apoptosis [26]. We expressed 30A-GAL4>G-trace in this background, and saw a reduction in fate change and translocation by the hinge cells (Fig 5, compare F to B, quantified in Fig 5M). We conclude that caspase activity and/or cell death is required for IR-induced regenerative behavior of the hinge cells. H99 heterozygosity also prevented ectopic disc formation in irradiated discs (Fig 5O), indicating that IR-induced apoptosis/caspase activity is also needed for the formation of ectopic discs.

### The requirement for effector caspase activity is in regenerative cells

H99 deficiency reduces apoptosis and caspase activity throughout the disc. Prior studies of regeneration in larval wing discs showed that apical caspase activity is needed in the dying cells to stimulate the neighbors to proliferate [24]. But no study we are aware of has addressed the need for caspase activity in the regenerative cells. Yet, there is mounting evidence for the role of caspases in cell fate changes [27, 28]. Our identification of regenerative cells in the hinge and the ability to target UAS-transgenes using 30A-GAL4 allowed us to address this possibility. We found that 30A-GAL4-driven expression of UAS-p35, an inhibitor of effector caspases, inhibited the translocation and fate change of the hinge cells (Fig 5, compare H to B, quantified in Fig 5M). 30A>p35 also inhibited the formation of ectopic discs (Fig 5O). We conclude that effector caspase activity is needed cell-autonomously (within the red cells in Fig 5N) for IR-induced regenerative behavior.

**Fig 6:**
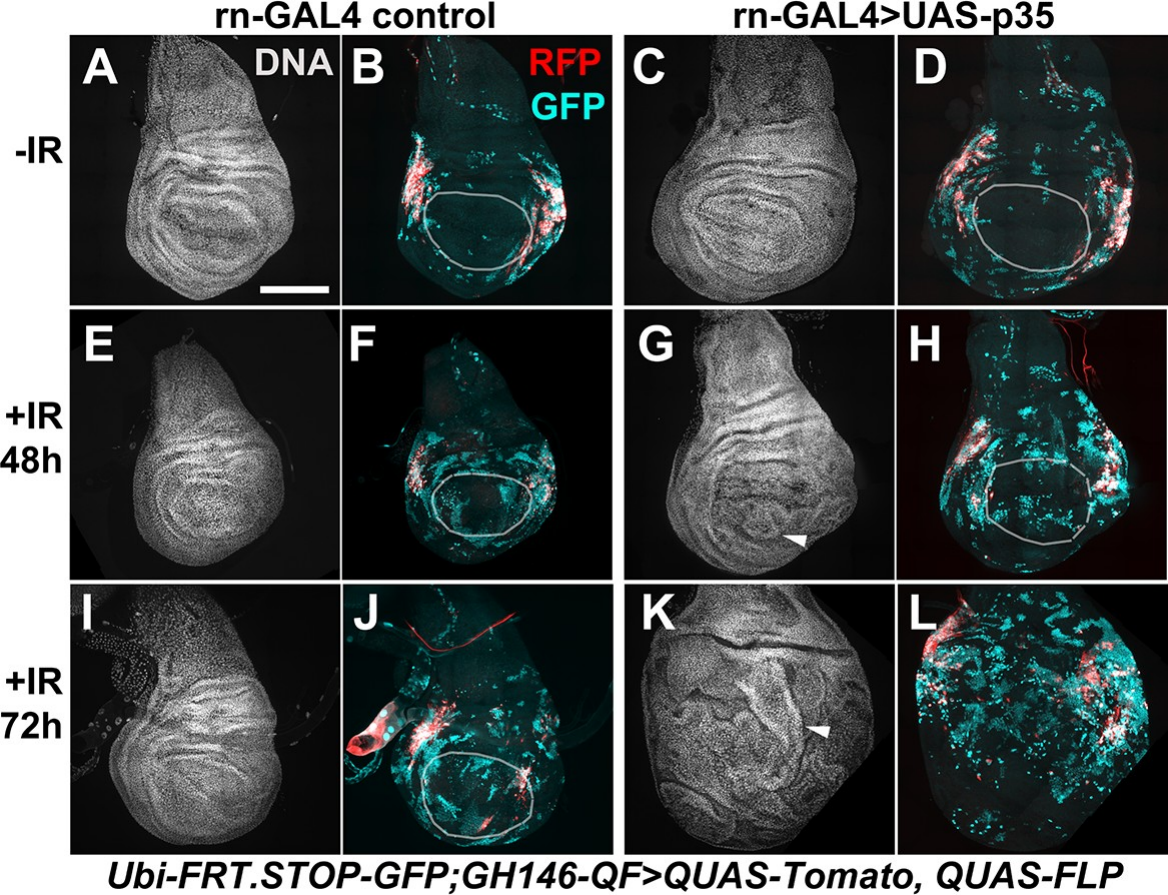
p35 in the pouch did not interfere with fate change and translocation of the hinge cells. The embryos were collected at 25°C for 24 h and cultured at 25°C throughout because there was no GAL80^ts^ in these experiments. The larvae were irradiated at 4–5 d after egg laying with 0 or 4000 R of X-rays. -IR samples were dissected 24 h after (mock) irradiation and +IR samples were dissected at times indicated on the figure, fixed and stained for DNA and imaged for RFP/GFP. DNA images were used to locate the pouch when possible (white circles). The discs are shown anterior left and dorsal up when discernable; in irradiated discs, p35 expression results in ‘undead’ cells that overgrow into folded layers (arrowheads), making it difficult to locate the pouch or disc orientation. Scale bar = 100 microns. The genotypes were: (A-B, E-F, I-J) rn-GAL4 control = P{w[+mC] = Ubi-p63E(FRT.STOP)Stinger}9F6/ P{w[+mC] = QUAS-FLPo.P}1; PBac{Disc\RFP[DsRed2.3xP3] = GH146-QF.P}53 P{w[+mC] = QUAS-mtdTomato-3xHA}24A, rn-GAL4/TM6-TB (C-D, G-H, K-L) rn-GAL4>UAS-p35 = P{w[+mC] = Ubi-p63E(FRT.STOP)Stinger}9F6/ P{w[+mC] = QUAS-FLPo.P}1; PBac{Disc\RFP[DsRed2.3xP3] = GH146-QF.P}53 P{w[+mC] = QUAS-mtdTomato-3xHA}24A, rn-GAL4/UAS-p35.

In reciprocal experiments, we expressed p35 in the pouch, to ask if effector caspase activity is needed in the pouch for the hinge to change fate and translocate. This is of interest because we and others have shown that dying cells secrete signals that change the behavior of surviving cells, including mitogenic signals [24] and ‘do not die’ signals [29]. We followed an experimental set-up used previously to show that when pouch cells were killed by rn-GAL4>UAS-*hid*, a pro-apoptotic gene, regeneration occurred with cells that immigrated from the hinge ([30]; the role of caspases were not addressed in this study). In these experiments, because GAL4>UAS was used in the pouch, the hinge cells were marked using the orthologous QF>QUAS system. We used the same GH146-QF driver, which is active in two pockets of hinge cells ([30]; see also Fig 6A and 6B), to mark the hinge, rn-GAL4 to express p35 in the pouch, and induced cell killing by IR (Fig 6). We found that GFP^+^ former hinge cells translocated into the pouch but only in irradiated discs (compare Fig 6B to 6F and 6J). p35 inhibits effector caspases but not initiator caspases in *Drosophila* [31, 32]; expression of p35 in the context of apoptosis induction results in ‘undead’ cells that initiate but cannot complete the apoptosis program and instead produces tissue overgrowth and disorganization [33]. We saw such overgrowth in the pouch of p35 expressing cells in the context of IR-induced apoptosis (arrowheads in Fig 6G and 6K). It was possible to discern the pouch using DNA staining at 48 h after IR but not at 72 h after IR because of overgrowth. Regardless, we observed GFP^+^ cells in the pouch (Fig 6H) or the pouch area (Fig 6K and 6L), and conclude that effector caspase activity is not needed in the pouch for the hinge cells to lose RFP and translocate.

### The requirement for apical caspase and JNK activity in regenerative cells

Apical caspase Dronc is needed to activate effector caspases [34] and is required for X-ray induced apoptosis in the wing [35] and the eye [36] discs. We co-expressed a catalytically inactive C->A mutant (UAS-proDronc^DN^) [37], and found that both fate change and translocation of hinge cells and ectopic disc formation were inhibited to similar extent as did H99/+ and p35 (Fig 5I and 5J, quantified in M and O).

Dronc, together with stress-responsive JNK kinase, also functions within dying cells for mitogenic signaling [24]. This function of Dronc is considered separable from its function in activating effector caspases. In the rn-GAL4>*hid* model described above, inhibition of JNK in the pouch by overexpression of phosphatase Puc inhibited the immigration of hinge cells into the pouch [30]. We performed a reciprocal experiment to ask if JNK activity was needed in the hinge in our IR-based model. Co-expression of UAS-Puc with 30A-GAL4>G-trace had a statistically significant effect on the translocation of the hinge cells (Fig 5K and 5L, quantified in Fig 5M). But the effect was minor; normalized GFP+RFP- area decreased from 0.56±0.14 in ‘*w*^*1118*^+IR’ to 0.36±22 in ‘puc+IR’, p = 0.02). In contrast, puc had a stronger inhibitory effect on the formation of ectopic discs (Fig 5O). This is similar to our previous finding that depletion of nucleosome remodeling factor Nurf-38 with 30A-GAL4>RNAi had a greater effect on ectopic disc formation than on hinge translocation [14]. We had reported that while ectopic disc formation requires a temperature shift (irradiated larvae kept at 25°C throughout the experiment do not form ectopic discs; [14]), a temperature shift is not necessary for the hinge-to-pouch regeneration [13], further distinguishing the two modes of regeneration.

### Zfh2 acts in the hinge to prevent apoptosis and to promote regenerative behavior

**Fig 7:**
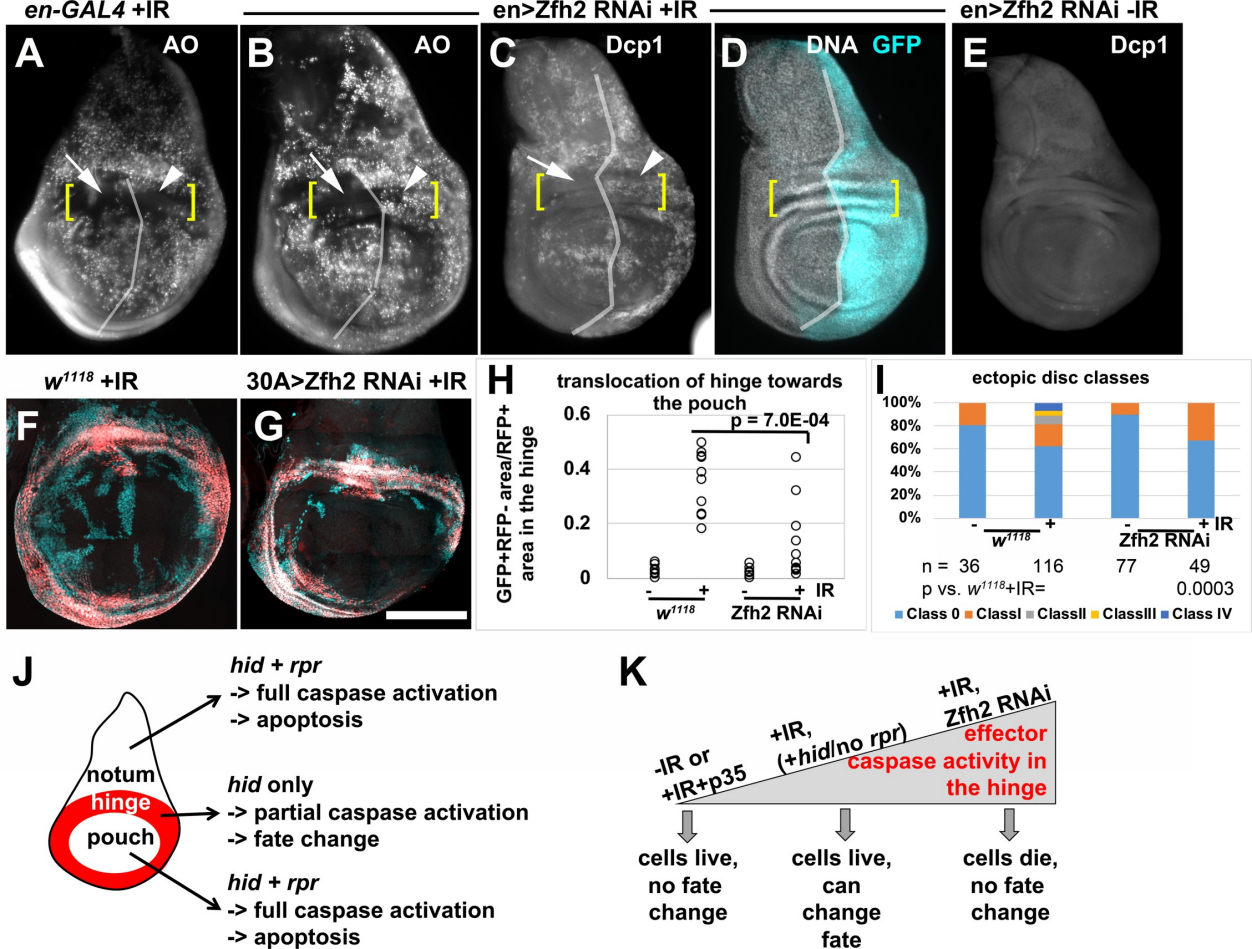
Zfh2 is needed in the hinge to prevent apoptosis and promote regenerative behavior. (A-E) To test the effect of Zfh2 depletion on IR-induced apoptosis, embryos were collected for 24 h at 25°C and cultured at 25°C until 3–4 d after egg laying. The larvae were shifted to 29°C to initiate RNAi and allowed to deplete Zfh2 for 48 h before irradiation with 0 or 4000R of X-rays. The discs were dissected 4 h after IR, and stained live with Acridine Orange (A-B) or fixed and stained with an antibody against cleaved Dcp1 (C-E). Fixed discs were also stained for DNA and imaged for GFP. In (C-D) en>GFP was used to locate the A/P boundary (vertical line) and the DNA stain was used to locate the dorsal hinge (brackets). In A-B, the A/P boundary and the dorsal hinge were estimated. Scale bar = 100 microns. (F-G) To test the effect of Zfh2 depletion on the behavior of the hinge, larvae were treated as in (A-E) except for being irradiated with 0 (-IR) or 4000 R (+IR) of X-rays 24 h after the shift to 29°C, and dissected 48 h (for -IR) or 72 h (for +IR) after irradiation. (H) Translocation of hinge cells was quantified from discs such as those in (F-G), as described in Fig 5. n = 10–11 each in two biological replicate experiments. Statistical significance was determined using 2-tailed student’s t-test. (I) Ectopic discs classes as determined from experiments shown in (F-G), as described in Fig 5. The number of discs examined for each genotype (n) is shown along with the p-value vs. *w*^*1118*^+IR, by Fisher Exact Test (see Methods). (J) A schematic diagram to show the difference in pro-apoptotic gene expression and apoptosis in the three main parts of the wing disc. (K) A schematic diagram to show how different levels of effector caspase activity could be generated within the hinge, along with the corresponding cell behavior. Scale bar = 120 microns in (A-E) and 100 microns in (F-G). The genotypes were: (A) en-GAL4, UAS-GFP/+; tub-GAL80^ts^/SM6-TM6B TB (B-E) en-GAl4, UASGFP/UAS-Zfh2 RNAi; tub-GAL80^ts^/+ (F) *w*^*1118*^ = 30A-GAL4, UAS-G-trace/+; tub-GAL80^ts^/+, from a cross of *w*^*1118*^ to 30A-GAL4, UAS-G-trace/CyO-GFP; tub-GAL80^ts^/ tub-GAL80^ts^ (G) 30A-GAL4, UAS-G-trace/UAS-Zfh2 RNAi; tub-GAL80^ts^/+.

Zfh2 is a transcription factor and a downstream effector of JAK/STAT signaling during wing development [38]. In addition, Zfh2 has been shown to prevent apoptosis in tissues under stress [39]. During normal development, Zfh2 expression is confined to the hinge in the 3^rd^ instar wing disc (Fig 1K). We published before that in irradiated discs, the hinge is protected from apoptosis ([13]; Fig 7A, brackets indicate the dorsal hinge). Therefore, we asked if Zfh2 has a role in preventing IR-induced apoptosis in the hinge. Zfh2 is required for wing hinge development [22, 38]. To deplete it we followed a protocol, described in Fig 7 legend, which is similar to what we used in published studies to deplete STAT and Wg that are also required for hinge development [14]. Zfh2 was conditionally depleted by en-GAL4-driven RNAi in the posterior half of the disc while the anterior half served as an internal control. We found that this treatment increased IR-induced apoptosis specifically in the posterior hinge (Fig 7B and 7C, compare arrows to arrowheads; brackets indicate the dorsal hinge). This increase was detected using Acridine Orange, which is excluded from live cells but penetrates dying cells [40, 41], and by antibody staining for cleaved caspase Dcp-1. Without Zfh2 RNAi, cell death was similar in anterior and posterior hinge (Fig 7A) and Zfh2 RNAi did not induce apoptosis without IR (Fig 7E). We conclude that Zfh2 is needed to protect the hinge cells from IR-induced apoptosis.

We next investigated the consequences of conditional Zfh2 depletion on the regenerative behavior of hinge cells, using the 30A-GAL4>G-trace (Fig 7F and 7G). Similarly treated *w*^*1118*^ controls show the translocation of hinge cells towards the pouch (Fig 7F, quantified in H). Depletion of Zfh2 inhibited the appearance of GFP^+^RFP^-^ cells in the pouch area (Fig 7G, quantified in H). Collectively, these data suggest a cell-autonomous requirement for Zfh2 in the hinge to temper IR-induced apoptosis and to promote IR-induced fate change and translocation. As in the case of H99/+ and p35, 30A-GAL4>Zfh2 RNAi also prevented ectopic disc formation (Fig 7I).

Our published data offer an explanation for how the hinge is protected from IR-induced caspase activation and apoptosis [13]. After irradiation, *hid* mRNA increases throughout the disc. *rpr* is transcriptionally induced only in the notum and the pouch and not in the hinge where it is repressed by Wg. Thus, while the cells in the notum and the pouch experience increased *hid* and *rpr* and undergo apoptosis, only *hid* is induced in the hinge and appears insufficient for apoptosis ([13], summarized in Fig 7J). These published data allow us to interpret the results reported here (modeled in Fig 7K). During normal development (-IR), cells of the hinge lack effector caspase activity and do not die, change fate, or translocate. The same situation applies in irradiated discs expressing 30A-GAL4>p35 (+IR+p35). After irradiation (+IR), *hid* is induced in the hinge but not *rpr*, which we propose leads to caspase activity that is insufficient for apoptosis but sufficient for fate change and translocation; 30A-GAL4>p35 blocks this intermediate caspase activity to inhibit regenerative behavior in irradiated discs. Upon depletion of Zfh2 in the hinge (this report) or inhibition of STAT/Wg activity [13], IR induces sufficient caspase activity, which is compatible with apoptosis but not with fate change and translocation. Thus, Zfh2 serves to keep alive cells with radiation stress and a low level of effector caspase activity, thereby allowing them to adopt new fate and location. This is in agreement with the report that Zfh2 is needed to keep alive JNK-active regenerative cells in a genetic ablation model in the wing disc [39]. Similar regulatory mechanisms may apply in the irradiated notum but with a lot fewer cells displaying intermediate effector caspase activity, which can explain why ectopic discs form only in a small fraction of irradiated discs.

**Fig 8:**
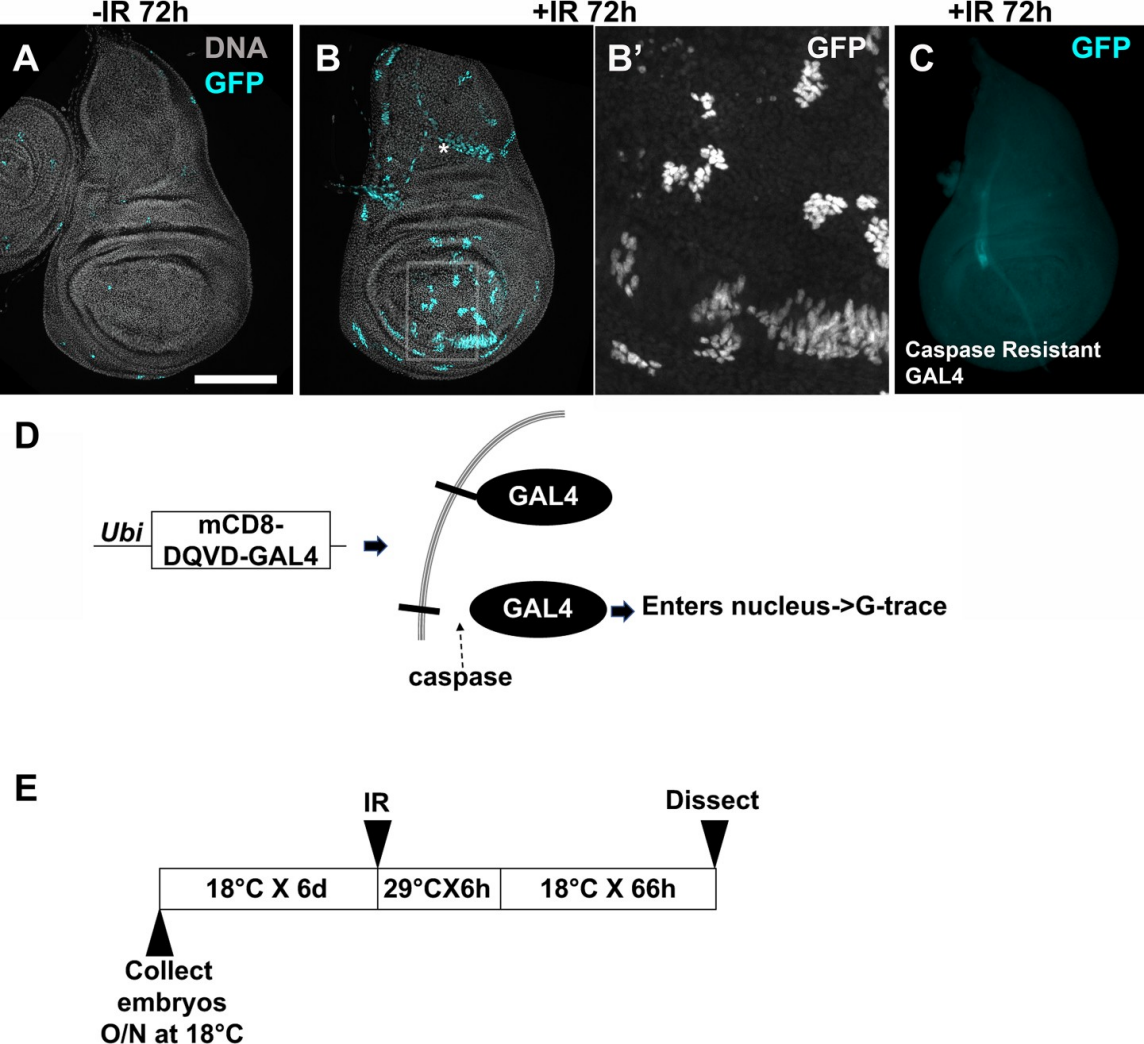
Regenerated wing discs include cells that activated caspases but did not die. (A-C) Larvae were cultured as in the protocol in (E) and irradiated with 0 (-IR) or 4000R (+IR) of X-rays. Wing discs were dissected 72 h after IR, fixed and stained for DNA and imaged for GFP. *in (B) marks large nuclei outside the plane of the columnar epithelial layer that show active CaspaseTracker (see also S2 Fig). The boxed area in (B) is magnified 4X and shown in (B’). The larvae in (C) expressed a control in which the caspase cleavage site in the membrane tether was mutated to DQVA to render it caspase-resistant. 19 ‘-IR’ and 20 ‘+IR’ discs for CaspaseTracker and 46 ‘+IR’ discs for caspase-resistant control were analyzed in two independent experiments. Scale bar = 25 microns in B’ and 100 microns in other panels. (D) A schematic diagram to explain CaspaseTracker. (E) The temperature shift protocol used to activate CaspaseTracker for 6 h after irradiation. CaspaseTracker; GAL80^ts^ embryos were collected and the larvae cultured at 18°C until irradiation. Irradiated larvae were incubated at 29°C for 6 hr to inactivate GAL80, then shifted back to 18°C for the remainder of the experiment. The genotypes were: (A-B) G-trace/CaspaseTracker GAL4; GAL80^ts^/+. (C) G-Trace/Caspase resistant-GAL4; GAL80^ts^/+.

One prediction of the above model is that an irradiated wing disc contains cells that activated caspases but did not die and instead contributed to the regenerated disc. To test this prediction, we used ‘CaspaseTracker biosensor’, which is a membrane-tethered GAL4 that is ubiquitously expressed in all cells in *Drosophila* [42, 43]. In the presence of active effector caspases, the tether is cleaved at DQVD to release GAL4, which enters the nucleus to activate G-trace (Fig 8D). CaspaseTracker>RFP is not a real time reporter of caspase activity because it takes several hours to express [42], but CaspaseTracker>GFP lineage expression effectively marks cells that activated caspases but did not die. The sensitivity of CaspaseTracker is such that there is a substantial number of GFP^+^ cells without IR or without GAL80^ts^ [43]. Therefore, we used GAL80^ts^ and optimized the conditions to express CaspaseTracker within a narrow (6 h) window immediately following irradiation (Fig 8E). This protocol produced very few GFP^+^ cells in unirradiated discs (Fig 8A), and allowed us to detect changes in irradiated discs (Fig 8B). Caspase-resistant controls in which DQVD has been mutated to DQVA [42] do not show GFP^+^ cells even with irradiation (Fig 8C), indicating that we are capturing caspase activity. Clusters of CaspaseTracker>GFP^+^ cells were found throughout the irradiated disc (Fig 8B). We do not know how their origin is spatially distributed within the disc; addressing this issue will require a non-FLP/FRT-based lineage tracing system for use in conjunction with CaspaseTracker, which we plan to develop in the future. Most GFP^+^ clusters were composed of at least 8 cells each (Fig 8B’), supporting the idea that irradiated discs include cells that activated effector caspases but did not die and instead went through at least three cell cycles.

## Discussion

In tumor biology, the concept of cancer stem cells has been controversial, but there is agreement that within a tumor, some cancer cells are better than others at re-initiating tumor growth [15, 16]. Eradication of ‘Cancer Stem-like Cells’ (CSCs) is considered necessary for successful therapy. However, not only do CSCs generate non-stem cancer cells, non-stem cancer cells also are capable of converting to CSCs. Even more concerning, cancer treatments including IR converts non-stem cancer cells from a variety of cancer types into cells with CSC markers that can initiate new tumors in culture and in vivo [19–21]. An estimated 50% of cancer patients receive IR, alone or as part of their treatment (www.cancer.org). Yet, we know very little about what aspects of IR exposure induce CSCs. The finding that IR induces non-stem cells of the *Drosophila* larval wing discs to exhibit stem cell like properties allowed us to fill in the gaps in this knowledge.

### Rules for IR-induced regenerative behavior

This study identified multiple specific pools of cells that show IR-induced changes in cell fate and location. A subset of hinge cells can do so, to become part of the regenerated pouch in nearly all irradiated discs. A different subset of cells in the hinge, together with cells of the pleura and the notum, produce an ectopic disc in about a tenth of irradiated discs. There are, however, limits to fate changes. For example, we observed very little disruption of A/P and D/V boundaries in the primary hinge, which means hinge cells that changed fate and translocated remained in their original quadrant. The A/P boundary is already established and the ventral marker Ap is already active in early 2^nd^ instar disc when the distinction between the hinge and the pouch is yet to be made [44]. Thus, the hinge-to-pouch conversion we detect reflects a developmentally more recent fate choice than A/P or D/V choices, identifying a limitation in IR-induced fate change. The fluidity of the A/P boundary in ectopic discs (S4 Fig), together with notum-to-pouch conversion in their generation suggest that fate changes during ectopic disc formation reflects events further back in development. But ectopic disc formation is rare and requires a temperature shift protocol, again illustrating that IR-induced fate change is not limitless. In another example, cells of the pouch display little indication that they change fate or translocate (S3 Fig) and not even when we directed cell death to the hinge and left the pouch cells alive [13]. This parallels how IR induces Cancer Stem Cell-like properties in some cancer cells but not others. Understanding how IR-induced fate changes are limited would be an important future goal.

### Comparison of our results to the literature

There are several insightful reports of regeneration after genetic ablation where cell death is directed to a specific compartment of the wing disc (for example, [45–48]). Different models of regeneration including ours rely on common factors such as STAT and Wg, but also show different molecular requirements. For example, heterozygotes of transcriptional factor CtBP increased the incidence of ectopic discs in a genetic ablation model [48]. But the same alleles of CtBP, we found, did not increase IR-induced ectopic discs or hinge-to-pouch fate change and translocation (S5 Fig). Even within genetic ablation models, the choice of apoptotic gene used to kill cells can have different outcomes on the regenerative behavior of the surviving cells. For example, ectopic discs formed in the notum when the pouch was ablated with Eiger (*Drosophila* TNF) but not with Rpr [48].

A recent study used genetic ablation to probe the regenerative potential of the notum. After ablation of the pouch and the hinge, the notum showed no increase in proliferation, did not regenerate the hinge or the pouch, and instead duplicated itself. The authors concluded that the notum cells have little regenerative potential [46]. This is in sharp contrast to IR-induced regenerative behavior we see in the notum. After IR, we detect a 3-fold increase in mitotic activity in the notum [14], and lineage tracing shows the notum contributes to the ectopic discs (this report). Our results parallel more closely what happens after genetic ablation of the pouch, which resulted in the production of ectopic wing discs in some mutant backgrounds [48]. Lineage tracing suggested that notum cells changed fate to contribute to the ectopic disc in that model, which agrees with our results. We add to this picture by identifying additional pools of cells in the dorsal-posterior hinge/pleura that contribute to the ectopic discs (Fig 4E).

Collectively, these data illustrate how different regenerative models rely on different molecules and cellular behaviors, hence the need to study each to learn the range of possibilities. This, we believe, is particularly true in the case of IR where damage (i.e. cell death) is not confined to any particular compartment but scattered throughout the wing disc in a reproducible pattern [13].

### Caspases link IR to regenerative behavior

One key gap concerns the question ‘what are the consequences of IR that induce stem cell-like behavior?’ The answer, we report here, is caspase activity. Surprisingly, we detect this requirement in regenerative cells that translocate and change fate. We propose that the outcome depends on the extent of effector caspase activity as modeled in Fig 7K. Regeneration typically relies on surviving cells proliferating and re-programming to replace cells lost to cell death or surgical removal. Prior work on *Drosophila* wing discs found that cell death itself induces Apoptosis-induced Proliferation in the surviving cells. In AiP, signaling through apical caspase Dronc and JNK in dying cells act through Dpp (TGF–β) and Wg to promote cell division in the surviving cells [33, 49–51]. The role of Dronc in AiP is in addition to its role in activating effector caspases and apoptosis. This and other similar mechanisms that operate in other larval discs explain the proliferative aspect of regeneration, but the re-programming aspect remained to be better understood. Our study fills the gap in the knowledge by identifying the role of caspases. We note a key difference between AiP and fate change/translocation. The former requires apical caspase Dronc but not effector caspases [24], while the latter requires both apical and effector caspases (this report).

The requirement for caspases we identified lies within the regenerative cell population, e.g. the hinge. This could be because a few apoptotic cells within this population stimulate others to display regenerative behavior. If such instigators exist, they must be very small in number because 30A-GAL4>p35 experiments showed no sign of overgrowth indicative of undead cells, even when they were readily visible in the pouch (Fig 6). Alternatively, caspases could play a non-apoptotic role within the regenerative cell population. There is precedent for non-apoptotic roles of effector caspases ([50, 52–54]; reviewed in [27]). In a particularly relevant study, effector caspase CED-3 in *C*. *elegans* was found to cleave cell fate determinant Lin-28, which is unrelated to apoptosis, in order to ensure that cell fate changes and developmental transitions occur normally [28]. The emerging view is that for a cell to change fate, it is insufficient to change only the transcriptional activity. Transcripts and proteins associated with the old fate must also be eliminated, and miRNAs and caspases work together to complete this task [28]. An intriguing hypothesis in *Drosophila* is that IR activates caspases that similarly down-regulate key fate determinants (to terminate the hinge identity, for example) and allow cell fate changes to reach completion. Validating this hypothesis will require the identification and functional analysis of caspase targets in this process.

## Materials and methods

### Drosophila stocks and methods

These stocks are described in Flybase: *w*^*1118*^, 30A- GAL4 (on Ch II, Bloomington stock# or BL37534), Ptub-GAL80^ts^ (on Ch III), *rn-GAL4* (on Ch III), *en-GAL4* (on Ch II), *ci-GAL4* (on Ch II), *ap-GAL4* (on Ch II), UAS-p35 (on Ch III). These stocks are described in publications: vgQ-lacZ [25], UAS-Zfh2 RNAi [22], UAS-Dronc^DN^ [37], UAS-puc [55], and CaspaseTracker and caspase resistant stocks [43]. The stock used for lineage tracing is also described in Flybase; w*; P{UAS-RedStinger}4, P{UAS-FLP.D}JD1, P{Ubi-p63E(FRT.STOP)Stinger}9F6 /CyO (BL28280). Genotypes for some BL stocks are in S1 Table and include FlyLight stocks [16]. Stocks to express QF and rn-GAL4 were generated by standard *Drosophila* recombinant protocols, using starting stocks listed in S1 Table.

### Larvae culture and irradiation

Larvae were raised on Nutri-Fly Bloomington Formula food (Genesee Scientific). The cultures were monitored daily for signs of crowding, typically seen as ‘dimples’ in the food surface as larvae try to increase the surface area for access to air. Cultures were split at the first sign of crowding. Larvae in food were placed in petri dishes and irradiated in a Faxitron Cabinet X-ray System Model RX-650 (Lincolnshire, IL) at 115 kv and 5.33 rad/sec.

### Antibody staining

Antibodies to Zfh2 (1:400, rat polyclonal, [56]), Ci (1:500, rat monoclonal 2A1, deposited into Developmental Biology Hybridoma Bank by R. Holmgren [57]), Nubbin (1:50, mouse monoclonal 2D4, deposited into Developmental Biology Hybridoma Bank by Michalis Averof), cleaved caspase Dcp1 (1:100, rabbit polyclonal, Cell Signaling #9578S) and fluorescently labelled secondary antibodies (1:200, Jackson) were used (see also S1 Table). In all experiments, wing discs were dissected in PBS, fixed in 4% para-formaldehyde in PBS for 30 min, and washed three times PBTx (0.1% Triton X-100). For antibody staining, the discs were washed in PBS instead of PBTx after the fixing step, permeabilized in PBTx with 0.5% Triton X-100 for 10 min and rinsed in PBTx. The discs were blocked in 5% Normal Goal Serum in PBTx for at least 30 min and incubated overnight at 4°C in primary antibody in block. The discs were rinsed thrice in PBTx and incubated in secondary antibody in block for 2 h at room temperature. Stained discs were washed in PBT. The discs were counter-stained with 10 μg/ml Hoechst33342 in PBTx for 2 min, washed 3 times, and mounted on glass slides in Fluoromount G (SouthernBiotech).

### Image analysis

With the exceptions noted below, the discs were imaged on a Perkin Elmers spinning disc confocal attached to a Nikon inverted microscope, using an SDC Andor iXon Ultra (DU-897) EM CCD camera. The NIS- Elements acquisition software’s large image stitching tool was used for the image capture. 15–20 z-sections 1 um apart were collected per disc. Sections that exclude the peripodial cells were collapsed using ‘maximum projection’ in Image J. The exceptions are images in Fig 7A–7E, Fig 8C and S1 Fig, which were acquired on a Leica DMR compound microscope using a Q-Imaging R6 CCD camera and Ocular software.

### Statistical analysis

For sample size justifications, we used a simplified resource equation from [58]; E = Total number of animals − Total number of groups, where E value of 10–20 is considered adequate. When we compare two groups (*w*^*1118*^ vs H99/+, for example), 6 per group or E = 11 would be adequate. All samples subjected to statistical analysis exceed this criterion. Two tailed student t-tests were used to analyze the fate change and translocation of the hinge (Fig 5M and Fig 7H, S5 Fig) and Fisher Exact Test was used to analyze ectopic disc formation (Fig 5O, Fig 7I and S5 Fig). In the latter application, IR-induced classes (II-IV) were binned together to compare the number of class 0-I discs and class II-IV discs for one condition against the same for another condition.

## Supporting information

S1 Fig Lineage tracing with FlyLight GAL4 drivers. Larvae were treated as in Fig 1M–IR. Wing discs are removed, fixed and imaged for RFP/GFP. All discs are shown with anterior left and dorsal up. Scale bar = 120 microns. The genotypes were: UAS-G-trace/+; tub-GAL80^ts^/GAL4. Bloomington stock number (BL) and Flylight construct number (R#) and the locus of origin for the enhancer are indicated on each panel. We also tested and found no RFP/GFP expression with *BH-1* R81E08 (BL40117) and *doc-1* R45H05 (BL46529), and weak RFP expression in the peripodium with unc5 R93E10 (BL48420). R85E08 (*salm*), R42A07 (*dve*) and R76A01 (*tup*) showed good overlap of RFP/GFP and were used in lineage tracing studies.
(PDF)

S2 Fig G-trace expression in cells outside the columnar epithelial layer. Wing discs were removed from 3^rd^ instar larvae expressing R73G07-GAL4>G-trace and treated as in Fig 1M-IR, fixed and imaged for GFP. The disc shown is the same as in Fig 2A–2D. Three optical sections illustrate peripodial cells (A, arrows), columnar epithelial cells (B, but also visible in other optical sections) and tracheal cells (C, arrow). The disc is shown with anterior left and dorsal up.
(PDF)

S3 Fig Cells of the pouch do not show regenerative properties after irradiation. Larvae of the genotype UAS-G-trace/+; GAL80^ts^/ rn-GAL4 (A-H), R42A07-GAL4 (I-L) or R85E08-GAL4 (M-P) were treated as in Fig 1M. Wing discs were removed, fixed and imaged for RFP/GFP. The discs were also stained for DNA. All discs are shown with anterior left and dorsal up. Scale bar = 100 microns.
(PDF)

S4 Fig Cells do not cross pre-existing compartment boundaries during regeneration after IR. To ask if compartment boundaries are breached during regeneration after IR damage, we expressed G-trace in the anterior, dorsal and posterior compartments of the wing disc using ci-, ap-, and en-GAL4, respectively. Larvae were treated as in Fig 1M. Wing discs are removed, fixed and stained for DNA, and imaged for RFP/GFP. The discs in O-P were also stained with an antibody to anterior compartment marker Ci. All discs are shown with anterior (a) left, posterior (p) right, and dorsal up. Total number of disc examined were 56 ci-IR, 22 ci+IR, 35 en-IR, 93 en+IR, 46 ap-IR, and 88 ap+IR. Scale bar = 40 microns (N-P) or 120 microns (other panels).
(A-L) GFP and RFP overlap within the primary disc with or without IR. We did observe small populations of cells that breach the boundary even without IR (for example, arrow in F).
(M-P) Compartment boundaries are fluid in the ectopic disc. For example, en-GAL4>RFP^+^ and RFP^-^ cells co-mingle (N), RFP^*-*^GFP^+^ cells that used to have the posterior identify but have lost it are present in the ectopic disc (arrow in N) and some GFP^+^ former posterior cells express the anterior marker Ci (circled in O-P). The genotypes were:
(A-D) UAS-G-trace/ci-GAL4; tub-GAL80^ts^/+
(E-H) UAS-G-trace/ap-GAL4; tub-GAL80^ts^/+
(I-P) UAS-G-trace/en-GAL4; tub-GAL80^ts^/+.
(PDF)

S5 Fig Mutations in CtBP do not increase IR-induced regenerative behavior. Larvae of the genotype UAS-G-trace/+; GAL80^ts^/CtBP were treated as in Fig 7F and 7G. Wing discs were removed, fixed and imaged for RFP/GFP. The discs were also stained for DNA. All discs are shown with anterior left and dorsal up. Hinge-to-pouch fate change and translocation (E) and ectopic disc formation (F) were quantified as described in Fig 5. The data for CtBP mutants are shown alongside the data for identically treated *w*^*1118*^ controls from Fig 7 shown again to allow direct comparison. n = 10–11 per genotype per condition. Scale bar = 100 microns. The genotypes were:
*w*^*1118*^ = 30A-GAL4, UAS-G-trace/+; tub-GAL80^ts^/+, from a cross of *w*^*1118*^ to 30A-GAL4, UAS-G-trace/CyO-GFP; tub-GAL80^ts^/ tub-GAL80^ts^
*Df CtBP/+* = 30A-GAL4, UAS-G-trace/+; tub-GAL80^ts^/Df
*CtBP*^*03463*^*/+* = 30A-GAL4, UAS-G-trace/+; tub-GAL80^ts^/*CtBP*^*03463*.^
(PDF)

S1 Table Key resources. (PDF)

S1 Data Raw numbers for graphs. (XLSX)
