# Supplementary figures and images for: Ionizing radiation induces stem cell-like properties in a caspase-dependent manner in Drosophila
Source: PLoS Genet. 2018 Nov 21;14(11):e1007659. doi: 10.1371/journal.pgen.1007659 (PMC6248896; doi:10.1371/journal.pgen.1007659)

**S1 Figure. Lineage tracing with Flylight GAL4 drivers**

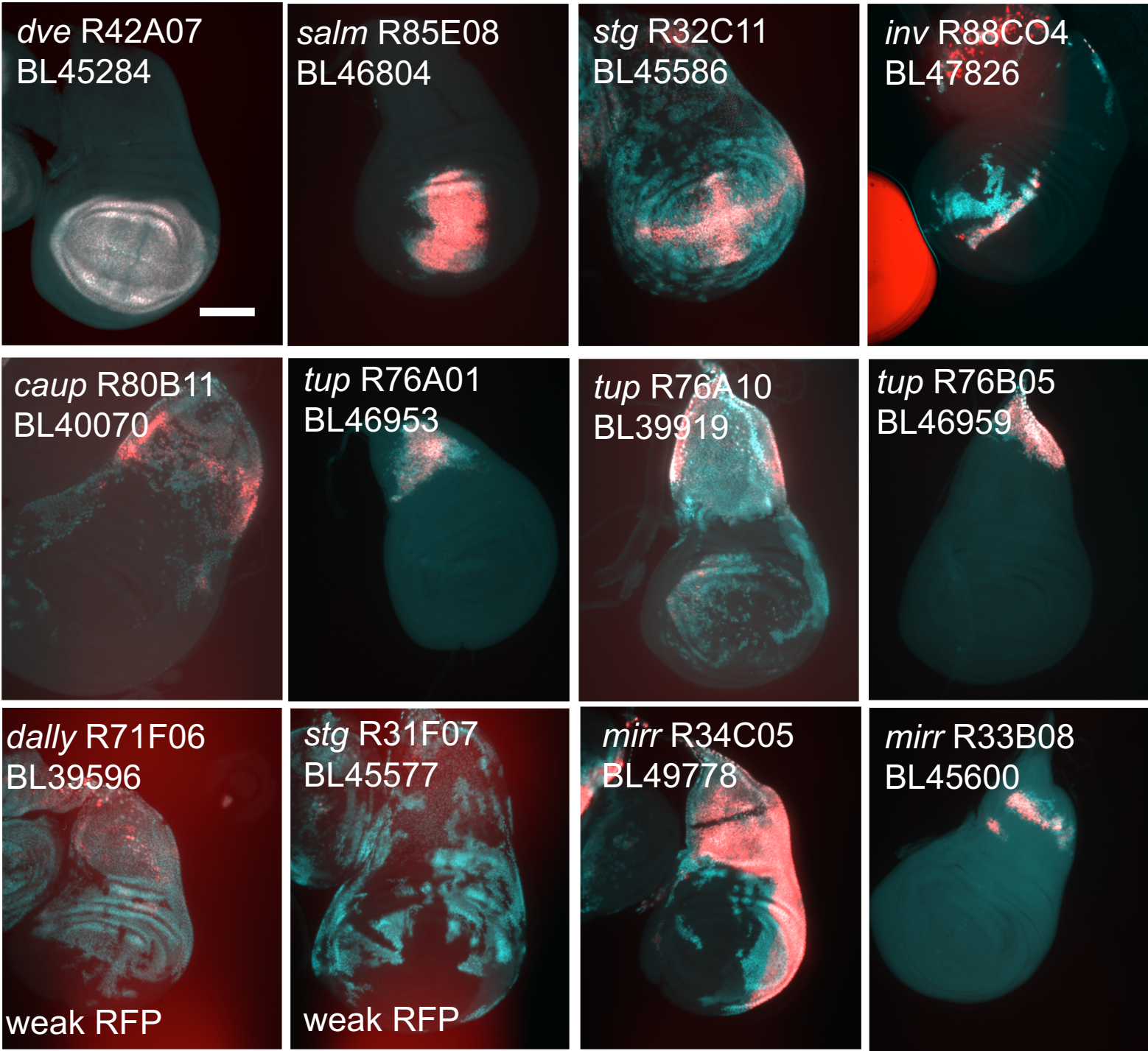

Supplement: S1 Fig — Larvae were treated as in Fig 1M–IR. Wing discs are removed, fixed and imaged for RFP/GFP. All discs are shown with anterior left and dorsal up. Scale bar = 120 microns. The genotypes were: UAS-G-trace/+; tub-GAL80ts/GAL4. Bloomington stock number (BL) and Flylight construct number (R#) and the locus of origin for the enhancer are indicated on each panel. We also tested and found no RFP/GFP expression with BH-1 R81E08 (BL40117) and doc-1 R45H05 (BL46529), and weak RFP expression in the peripodium with unc5 R93E10 (BL48420). R85E08 (salm), R42A07 (dve) and R76A01 (tup) showed good overlap of RFP/GFP and were used in lineage tracing studies. (PDF) [file pgen.1007659.s001.pdf]

**S2 Fig. G-trace expression in cells outside the columnar epithelial layer**

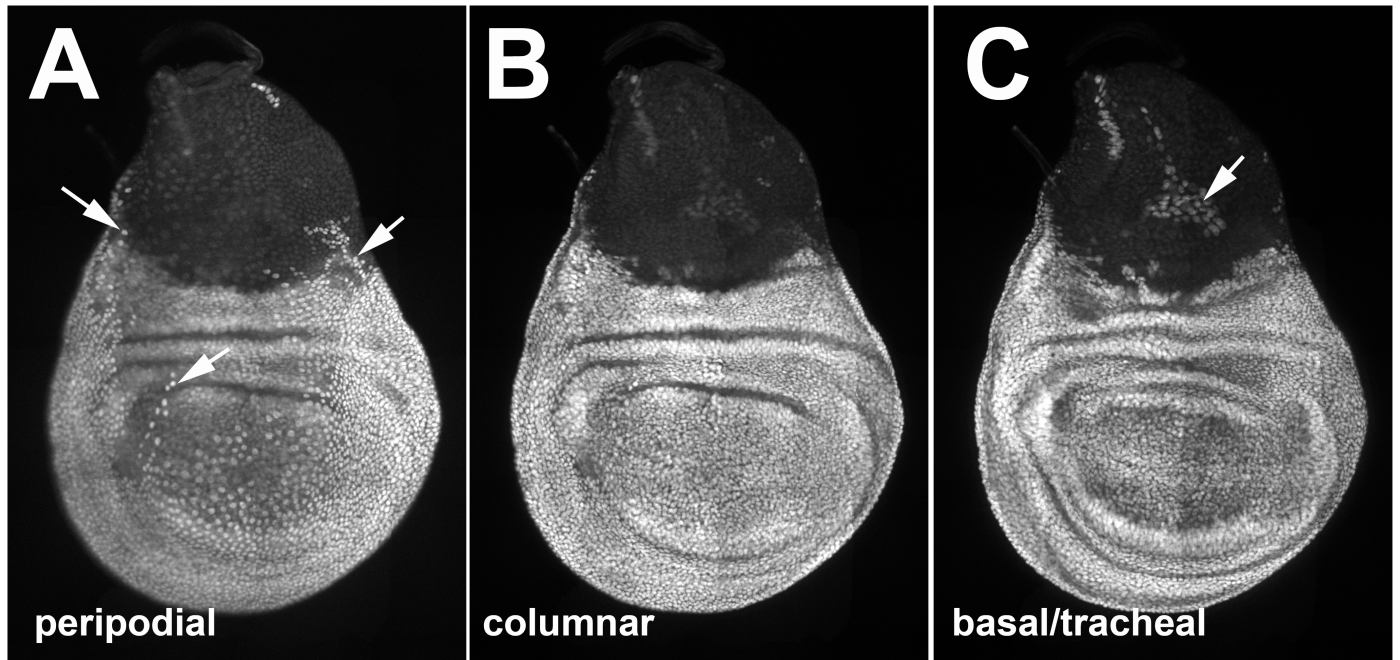

Supplement: S2 Fig — Wing discs were removed from 3rd instar larvae expressing R73G07-GAL4>G-trace and treated as in Fig 1M-IR, fixed and imaged for GFP. The disc shown is the same as in Fig 2A–2D. Three optical sections illustrate peripodial cells (A, arrows), columnar epithelial cells (B, but also visible in other optical sections) and tracheal cells (C, arrow). The disc is shown with anterior left and dorsal up. (PDF) [file pgen.1007659.s002.pdf]

S3 Fig. Cells of the pouch do not show regenerative properties after irradiation

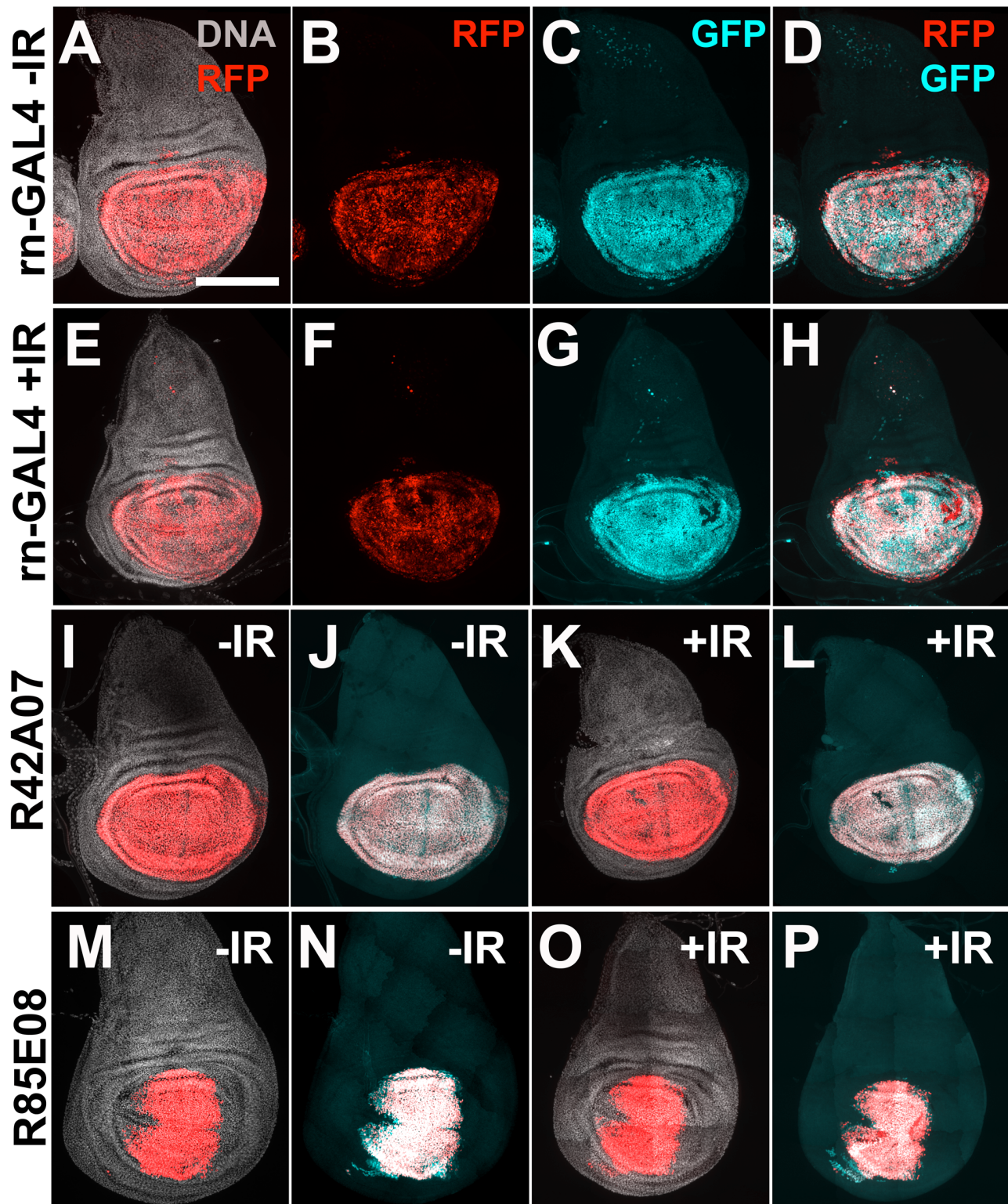

Supplement: S3 Fig — Larvae of the genotype UAS-G-trace/+; GAL80ts/ rn-GAL4 (A-H), R42A07-GAL4 (I-L) or R85E08-GAL4 (M-P) were treated as in Fig 1M. Wing discs were removed, fixed and imaged for RFP/GFP. The discs were also stained for DNA. All discs are shown with anterior left and dorsal up. Scale bar = 100 microns. (PDF) [file pgen.1007659.s003.pdf]

S4 Fig. Cells do not cross pre-existing compartment boundaries during regeneration after IR

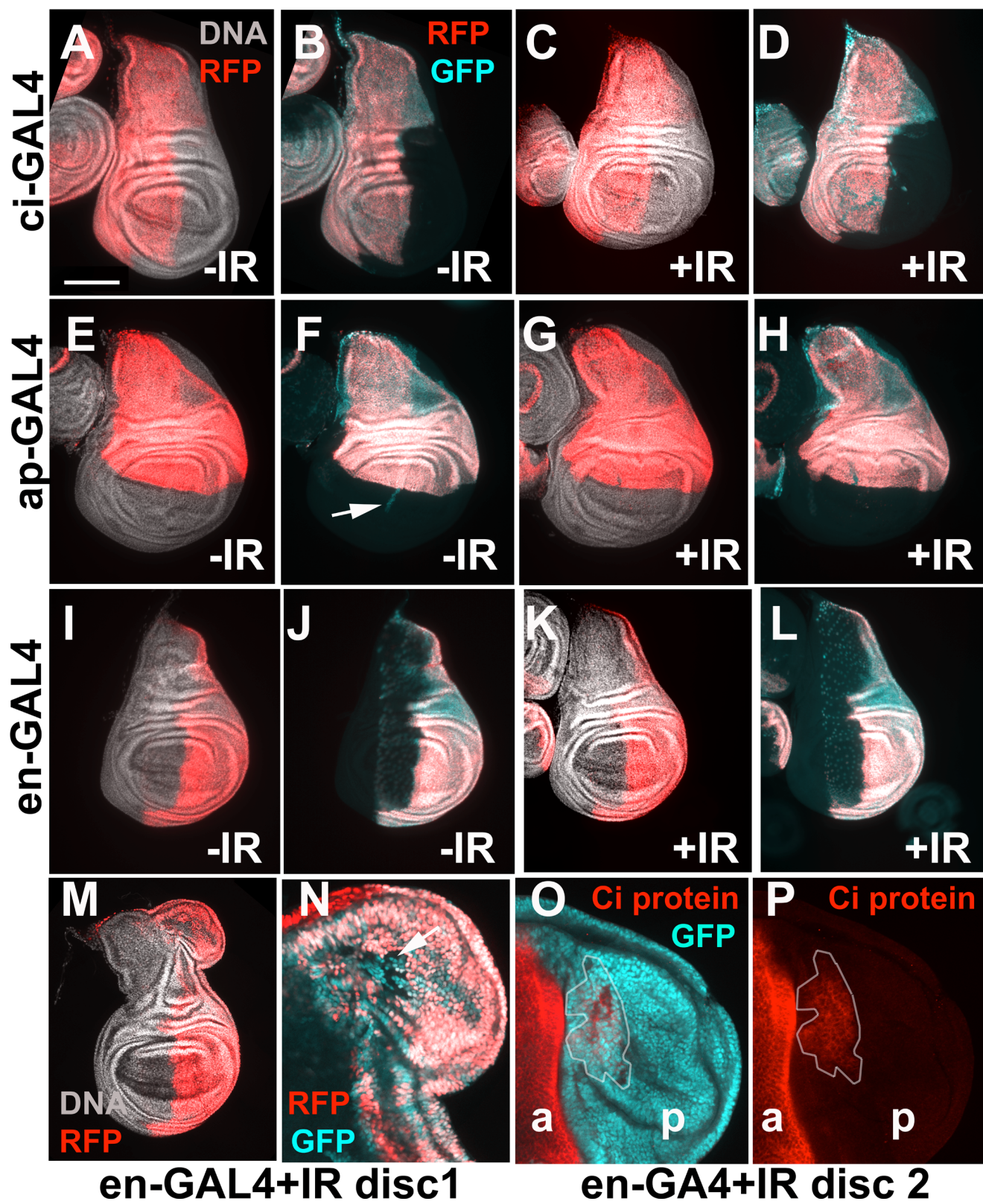

Supplement: S4 Fig — To ask if compartment boundaries are breached during regeneration after IR damage, we expressed G-trace in the anterior, dorsal and posterior compartments of the wing disc using ci-, ap-, and en-GAL4, respectively. Larvae were treated as in Fig 1M. Wing discs are removed, fixed and stained for DNA, and imaged for RFP/GFP. The discs in O-P were also stained with an antibody to anterior compartment marker Ci. All discs are shown with anterior (a) left, posterior (p) right, and dorsal up. Total number of disc examined were 56 ci-IR, 22 ci+IR, 35 en-IR, 93 en+IR, 46 ap-IR, and 88 ap+IR. Scale bar = 40 microns (N-P) or 120 microns (other panels). (A-L) GFP and RFP overlap within the primary disc with or without IR. We did observe small populations of cells that breach the boundary even without IR (for example, arrow in F). (M-P) Compartment boundaries are fluid in the ectopic disc. For example, en-GAL4>RFP+ and RFP- cells co-mingle (N), RFP-GFP+ cells that used to have the posterior identify but have lost it are present in the ectopic disc (arrow in N) and some GFP+ former posterior cells express the anterior marker Ci (circled in O-P). The genotypes were: (A-D) UAS-G-trace/ci-GAL4; tub-GAL80ts/+ (E-H) UAS-G-trace/ap-GAL4; tub-GAL80ts/+ (I-P) UAS-G-trace/en-GAL4; tub-GAL80ts/+. (PDF) [file pgen.1007659.s004.pdf]

S5 Fig. Mutations in CtBP do not increase IR-induced regenerative behavior

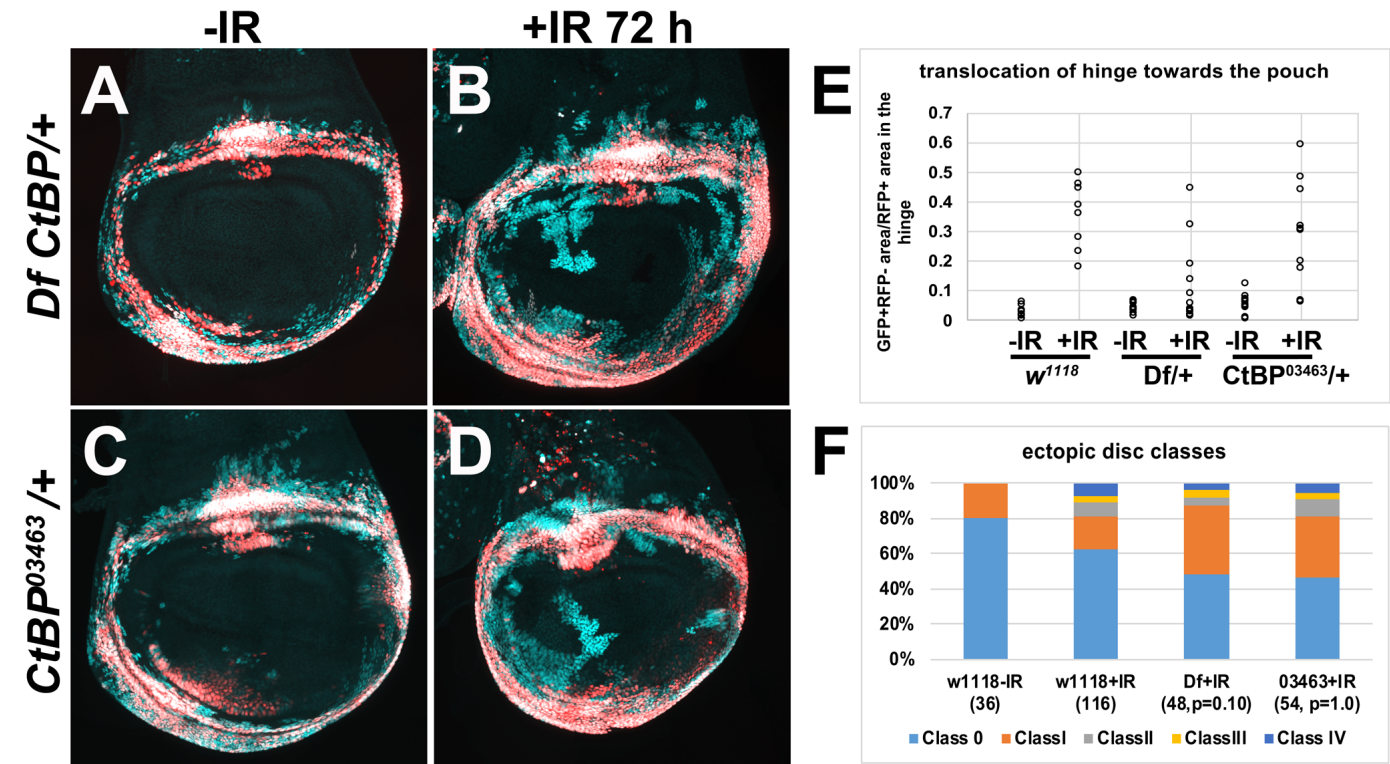

Supplement: S5 Fig — Larvae of the genotype UAS-G-trace/+; GAL80ts/CtBP were treated as in Fig 7F and 7G. Wing discs were removed, fixed and imaged for RFP/GFP. The discs were also stained for DNA. All discs are shown with anterior left and dorsal up. Hinge-to-pouch fate change and translocation (E) and ectopic disc formation (F) were quantified as described in Fig 5. The data for CtBP mutants are shown alongside the data for identically treated w1118 controls from Fig 7 shown again to allow direct comparison. n = 10–11 per genotype per condition. Scale bar = 100 microns. The genotypes were: w1118 = 30A-GAL4, UAS-G-trace/+; tub-GAL80ts/+, from a cross of w1118 to 30A-GAL4, UAS-G-trace/CyO-GFP; tub-GAL80ts/ tub-GAL80ts Df CtBP/+ = 30A-GAL4, UAS-G-trace/+; tub-GAL80ts/Df CtBP03463/+ = 30A-GAL4, UAS-G-trace/+; tub-GAL80ts/CtBP03463. (PDF) [file pgen.1007659.s005.pdf]
