# Supplementary material for: Ionizing radiation induces stem cell-like properties in a caspase-dependent manner in Drosophila
Source: PLoS Genet. 2018 Nov 21;14(11):e1007659. doi: 10.1371/journal.pgen.1007659 (PMC6248896; doi:10.1371/journal.pgen.1007659)
Supplement: S1 Table — (PDF) [file pgen.1007659.s006.pdf]

S1 Table. Key Resources

**Drosophila stocks**			
**Source (BL = Bloomington Stock Center)**	**Used in**	**Genotype**	**Associated locus (for FlyLight lines)**
BL 37534	Fig 1, 5, 7	w[*]; P{w[+mW.hs]=GawB}30A/CyO	
BL 28280 "G-trace"	Fig 1-5, 7	[*]; P{w[+mC]=UAS-RedStinger}4, P{w[+mC]=UAS-FLP.D}JD1, P{w[+mC]=Ubi-p63E(FRT.STOP)Stinger}9F6/CyO	
BL 39829	Fig 1-3, S2 Fig	w[1118]; P{y[+t7.7] w[+mC]=GMR73G07-GAL4}attP2	*zfh2*
BL 46953	Fig 4	w[1118]; P{y[+t7.7] w[+mC]=GMR76A10-GAL4}attP2	*tup*
BL 30564	Fig 7, S4 Fig	'en-GAL4' P{en2.4-GAL4}e16E	
[55]	Fig 5	UAS-puc on Chromosome III	
[25]	Fig 5	VgQ-lacZ/CyO	
BL 1576	Fig 5	Df(3L)H99/TM6B-TB	
BL 5073	Fig 5, 6	w[*]; P{w[+mC]=UAS-p35.H}BH2	
BL 30037	Fig 6	w[1118]; PBac{Disc\RFP[DsRed2.3xP3]=GH146-QF.P}53 P{w[+mC]=QUAS-mtdTomato-3xHA}24A	
BL 30126	Fig 6	w[1118]; P{w[+mC]=QUAS-FLPo.P}1	
BL 32250	Fig 6	w[*]; P{w[+mC]=Ubi-p63E(FRT.STOP)Stinger}9F6	
BL 7405	Fig 6, S3 Fig	'rn-GAL4' P{GawB}rnGAL4-5	
[22]	Fig 7	UAS-Zfh2 RNAi/SM6a-TM6B	
[43]	Fig 8	'CaspaseTracker' mCD8-DQVD or DQVA-Gal4/CyO	
BL 45284	S1, S3 Fig	w[1118]; P{y[+t7.7] w[+mC]=GMR42A07-GAL4}attP2	*dve*
BL 46804	S1, S3 Fig	w[1118]; P{y[+t7.7] w[+mC]=GMR85E08-GAL4}attP2/TM3, Sb[1]	*salm*
BL 45586	S1 Fig	w[1118]; P{y[+t7.7] w[+mC]=GMR32C11-GAL4}attP2	*stg*
BL 47825	S1 Fig	w[1118]; P{y[+t7.7] w[+mC]=GMR88A09-GAL4}attP2/TM3, Sb[1]	*inv*
BL 40070	S1 Fig	w[1118]; P{y[+t7.7] w[+mC]=GMR80B11-GAL4}attP2	*caup*
BL 39919	S1 Fig	w[1118]; P{y[+t7.7] w[+mC]=GMR76A10-GAL4}attP2	*tup*
BL 46959	S1 Fig	w[1118]; P{y[+t7.7] w[+mC]=GMR76B05-GAL4}attP2/TM3, Sb[1]	*tup*
BL 39596	S1 Fig	w[1118]; P{y[+t7.7] w[+mC]=GMR71F06-GAL4}attP2	*dally*
BL 45577	S1 Fig	w[1118]; P{y[+t7.7] w[+mC]=GMR31F07-GAL4}attP2	*stg*
BL 49778	S1 Fig	w[1118]; P{y[+t7.7] w[+mC]=GMR34C05-GAL4}attP2	*mirr*
BL 45600	S1 Fig	w[1118]; P{y[+t7.7] w[+mC]=GMR33B08-GAL4}attP2	*mirr*
	S3 Fig	ci-GAL4 on Chromosome II	
BL 3041	S4 Fig	'ap-GAL4' P{GawB}apmd544/CyO	
BL11590	S5 Fig	P{ry[+t7.2]=PZ}CtBP[03463] ry[506]/TM3, ry[RK] Sb[1] Ser[1]	
BL 7973	S5 Fig	'Df CtBP' w[1118]; Df(3R)Exel8157/TM6B, Tb[1]	

**Antibodies**			
**Source**	**Used in**	**Description**	**Identifier**
[56]	Fig 1	Rat anti Zfh2	N/A
Developmental Biology Hybridoma Bank	Fig 4	Mouse anti Nubbin	2D4
	S4 Fig	Rat anti Ci	2A1
Cell Signaling	Fig 7	Rabbit anti cleaved Dcp1	9578S
